# Cognitive genomics of learning delay and low level of social performance monitoring in macaque

**DOI:** 10.1038/s41598-022-20948-4

**Published:** 2022-10-03

**Authors:** Taihei Ninomiya, Atsushi Noritake, Shoji Tatsumoto, Yasuhiro Go, Masaki Isoda

**Affiliations:** 1 Division of Behavioral Development, Department of System Neuroscience, National Institute for Physiological Sciences, National Institutes of Natural Sciences, Okazaki, 444-8585 Japan; 2 Department of Physiological Sciences, School of Life Science, The Graduate University for Advanced Studies (SOKENDAI), Hayama, 240-0193 Japan; 3 Cognitive Genomics Research Group, Exploratory Research Center on Life and Living Systems (ExCELLS), National Institutes of Natural Sciences, Okazaki, 444-8585 Japan

**Keywords:** Genetics, Neuroscience, Physiology, Psychology

## Abstract

Cognitive skills and the underlying neural architecture are under the influence of genetics. Cognitive genomics research explores the triadic relationship between genes, brain, and cognition, with its major strategy being genotype-driven. Here we show that an inverse strategy is feasible to identify novel candidate genes for particular neuro-cognitive phenotypes in macaques. Two monkeys, originally involved in separate psychological studies, exhibited learning delay and low levels of social performance monitoring. In one monkey, mirror neurons were fewer compared to controls and mu suppression was absent in the frontal cortex. The other monkey showed heightened visual responsiveness in both frontal cortex and dopamine-rich midbrain, with a lack of inter-areal synchronization. Exome analyses revealed that the two monkeys were most likely cousins and shared variants in *MAP2*, *APOC1*, and potentially *HTR2C*. This phenotype-driven strategy in cognitive genomics provides a useful means to clarify the genetic basis of phenotypic variation and develop macaque models of neuropsychiatric disorders.

## Introduction

Cognitive skills and their underlying neural architecture are under genetic influence^1–4^. Clarifying the genomic basis of higher cognitive functions has been a longstanding interest in genome science. Recent advances in high-throughput technologies have made it possible, with significantly lower costs, to determine the whole-genome sequences of individuals. This advancement has led to the development of an interdisciplinary field of science called cognitive genomics^2,3,5^. Cognitive genomics research has the potential to determine the individual variation in cognitive traits in genetic terms, which in turn may not only help to develop animal models of mental disorders, but also promote an understanding of the evolutionary trajectories of gene-cognition associations.

Cognitive genomics research in nonhuman primates has mainly taken a ‘genotype-driven’ approach^6^. In this strategy, researchers predetermine the genes of interest and explore the cognitive aspects associated with the polymorphisms of the target genes. To date, several genes have been the target of study: *SLC6A4* (serotonin transporter) for cognitive flexibility^7^ and social gaze^8^ in macaques, *SLC6A4* and *TPH2* (tryptophan hydroxylase 2) for affiliative tendencies^9^ in macaques, and *AVPR1A* (arginine vasopressin receptor 1A) for sociality^10^ and mirror self-recognition^11^ in chimpanzees. Apart from genotype-driven approaches, an inverse ‘phenotype-driven’ approach is also feasible^6^. Herein, researchers focus on aspects of cognition, rather than genes, that vary between individuals and attempt to determine the genetic correlates of cognitive variation. This strategy can be applied to animals with unusual cognitive phenotypes, which may then be used as nonhuman primate models of neuropsychiatric disorders. Two rare coding variants have been identified in *ABCA13* and *HTR2C* in a Japanese macaque that spontaneously expressed the autistic phenotype^12^.

Here we report two Japanese macaques (M593 and M639) with substantial learning delays under laboratory conditions. Both monkeys also exhibited low levels of monitoring others’ performance despite differences in task conditions, i.e., a social reversal learning task (M593)^13^ and a social Pavlovian conditioning procedure (M639)^14^. Analyses of brain activity using microelectrodes revealed aspects of task-related responses in cortico-subcortical regions that were statistically different from their controls. Comprehensive genetic analysis via exome sequencing revealed kinship ties between the two monkeys (most likely cousins) and shared genetic variants in *MAP2*, *APOC1*, and possibly *HTR2C*. All of these genes have been linked to neuropsychiatric disorders in humans.

## Results

### M593: Behavioral profiles

M593 (*Macaca fuscata*, male, 5 years old at the beginning of experiments) was enrolled in the study of the neural basis of social action monitoring^13^. Behavioral abnormalities in M593 were not recognized in the home cage condition until behavioral training protocols were started. However, soon after the introduction of the pole-and-collar method for safe transfer of the animal from the home environment to a primate chair^15^, we realized that M593 was a slower learner. M593 needed as long as 5 months to become habituated to this training method, whereas other monkeys in our laboratory typically required 1–2 months.

**Figure 1:**
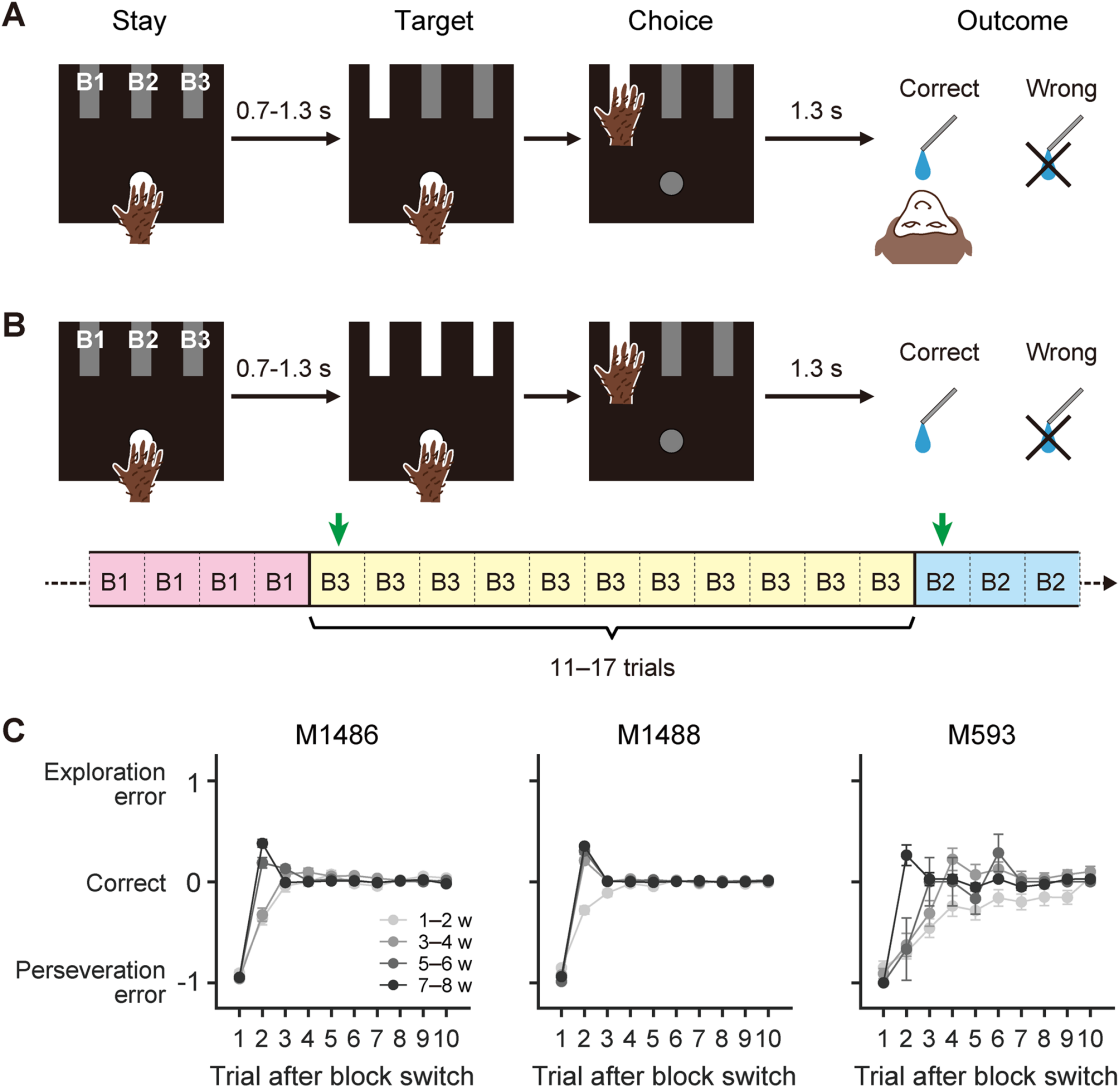
Behavioral procedures in non-social task condition for M593. (**A**) Sequence of events in visually-guided reaching task. (**B**) Sequence of events in non-social reversal learning task. The correct target was switched after every 11–17 trials without prior notice. Green arrows indicate switch trials. (**C**) Performance score in each trial after block switches. Mean ± SEM in Trial 1 (i.e., switch trial). Data are shown separately for different training phases. A score of − 1 was assigned when monkeys selected a target that was correct in the preceding block (perseveration error). A score of 0 was assigned when monkeys selected the correct target in the current block. A score of + 1 was assigned when monkeys selected a target that was incorrect in the previous and current blocks (exploration error).

Then, we installed an experimental device that consisted of a start button and three target buttons in the primate chair condition (Fig. 1A). M593 was trained to perform a reach-to-target movement in response to the onset of one of the three targets; M593 required 2 months to learn this simple, visually guided reaching task (typically monkeys take 1 week).

Then, behavioral training progressed to the next phase that was more specific to the current project. The three targets were illuminated simultaneously, and the monkey had to select only one of them by reaching toward it (Fig. 1B). The correct, rewarded target was fixed to one of the three targets for consecutive 11–17 trials (called a ‘block’), and then switched without prior notice to one of the two remaining targets in the next block (green arrows; Fig. 1B, bottom). During this training phase, the monkey was required to perform a reversal learning task.

In the same project, we tested two other monkeys (*M. fuscata*, males; M1486 and M1488) that were considered neurotypical. Their behavioral and neural data were reported previously^13^. M1486 and M1488 served as controls in the present study. Figure 1C shows the task performance progress of M593 and two control monkeys as they learned the reversal learning task. In the task, the choice in the first trial of each block (‘switch trial’) was considered to be wrong (i.e., unrewarded) because the block switch was not signaled in advance. Following this ‘switch error’, the monkeys had to switch to one of the two remaining targets in the second trial, rather than continuing to select the target that had been correct in the preceding block (‘perseveration error’). In the control monkeys, predominance of perseveration error in the second trial waned after 3 (M1488) or 5 (M1486) weeks of training. However, in M593, perseveration error was evident not only in the second trial but also in later trials, and waned after 7 weeks (Fig. 1C).

**Figure 2:**
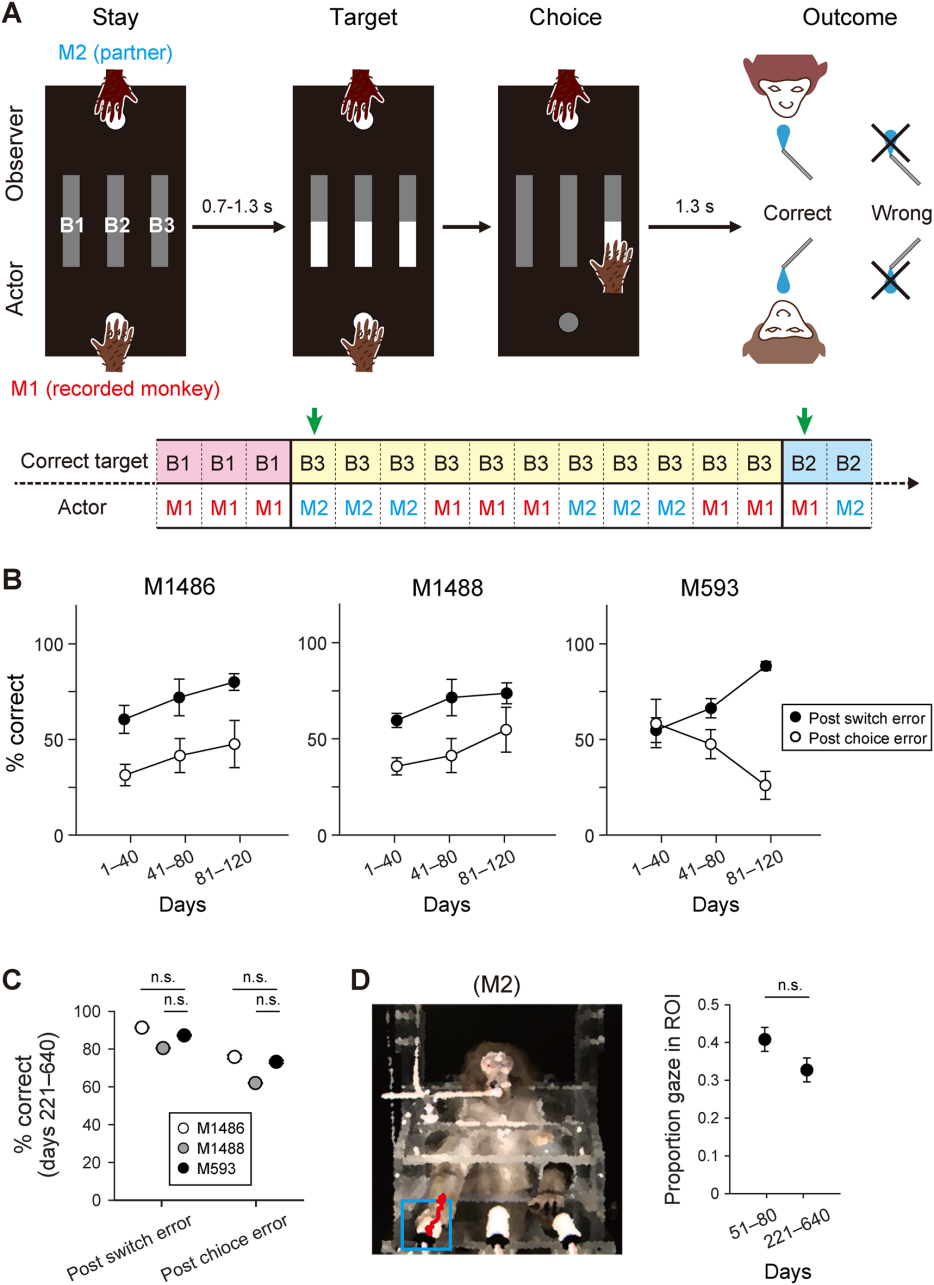
Behavioral procedures in social task condition for M593. (**A**) Sequence of events in social reversal learning task. M1, recorded monkey or self; M2, partner. The correct target was switched after every 11–17 trials without prior notice. Green arrows indicate switch trials. The actor role was alternated between M1 and M2 after every three trials. Reward feedback was presented to both monkeys simultaneously. (**B**) Time courses of performance accuracy in trials immediately after partner’s switch and choice errors. Mean ± SEM. Note the progressive divergence of performance in M593. (**C**) Performance accuracy at later learning stages (days 221–640). Mean ± SEM; n.s., not significant (two-tailed Welch’s *t*-test). (**D**) Gaze behavior. *Left*, blue rectangle indicates the target ROI when B1 was correct, and red dots indicate M1’s gaze direction. *Right*, proportions of gaze at the target ROI in earlier and later training days. Mean ± SEM; n.s., not significant (two-tailed Welch’s *t*-test).

Behavioral training was followed by the final stage. At this stage, a monkey partner was introduced face-to-face to the monkey being studied (Fig. 2A). The essence of this ‘social’ reversal learning task remained the same except for role assignment: in each trial, the roles of actor and observer were assigned to one monkey each. The actor role, which was indicated by illumination of the start button on the actor’s side, was alternated after every three trials. When the actor selected the correct target, both monkeys were rewarded; when the actor made an incorrect choice, neither monkey was rewarded. Therefore, both monkeys were informed of the correctness of the executed or observed actions.

In the social reversal learning task, M593 exhibited different learning trajectories from those of the control monkeys in terms of the manner in which the monkeys used the information about their partner’s choice in the preceding trial for their own choice in the current trial. To better illustrate this, consider two examples of the partner’s choices that result in a lack of reward. The first case occurred in non-switch trials, where the lack of reward was caused by the partner’s choice of an incorrect target (‘choice error’ case). The second case occurred in switch trials, where the lack of reward was caused by the partner’s selection of the previously correct target (‘switch error’ case). After these no-reward outcomes, the monkey being studied assumed the actor role and should keep selecting the correct target in the choice error case (i.e., exploitation because of the continuation of the same target-reward association), but should select different targets in the switch error case (i.e., exploration because of the change in the target-reward association). We found that the performance improved with practice in both error cases in the control monkeys (Fig. 2B, left and middle). However, in M593, the performance after the two error cases was gradually dissociated: improved performance in the switch error case, and deteriorated performance in the choice error case (Fig. 2B, right). Notably, this pattern of behavioral dissociation was observed previously in the autistic monkey M344^12^, as well as in the neurotypical monkey but with a selective blockade of the pathway from the ventral premotor cortex (PMv) to the medial prefrontal cortex (MPFC)^13^. Such behavioral dissociation can be accounted for by animals’ sticking to the win-stay lose-switch strategy, which is the most adaptive for the reversal learning task performed individually, but not adaptive in social settings^12,13^. These findings suggest that M593, but not M1486 and M1488, had difficulty in monitoring and/or using the partner action information. However, with prolonged practice, M593 eventually learned to perform the task comparably well to the control monkeys (Fig. 2C). Note that the behavioral dissociation was not explained by a mere decrease in attention to the partner’s choice (Fig. 2D).

### M593: Neuronal profiles

The brain magnetic resonance imaging (MRI) revealed no apparent structural abnormality in M593. To identify the neural correlate of behavioral properties of M593, we carried out multi-site, multi-electrode neural recordings from the PMv and MPFC, while M593 performed the social reversal learning task. Cortical recording sties were consistent between M593 and its controls (Fig. S1). As documented in the literature, the PMv and MPFC constitute the mirror system^16^ and mentalizing system^17^ in social brain networks, respectively. These cortical regions are two frontal nodes for social performance monitoring^18^.

Consistent with our previous report on the control monkeys^13^, three types of actor-related neurons were identified in M593: the ‘self type’ that responded selectively or preferentially to the self-action, the ‘mirror type’ that responded non-differentially to the self-action and partner-action, and the ‘partner type’ that responded selectively or preferentially to the partner-action (“Methods” for the definition of neuronal classification). In these neurons, the magnitude and latency of response to the action-target stimuli were not systematically different between M593 and its control monkeys (Fig. S2). However, the matrix obtained from principal component analysis revealed that M593 occupied distant positions in the principal space (Fig. S3A). We found that the proportion of mirror-type neurons among the total actor-related neurons was significantly lower in M593 compared to the control monkeys (Tables 1 and 2). The lower proportion of mirror-type neurons was confirmed in both PMv (Table 1; M593 vs. M1486, *P* = 0.004; M593 vs. M1488, *P* = 0.037; chi-square test) and MPFC (Table 2; M593 vs. M1486, *P* = 0.037; M593 vs. M1488, *P* = 0.038; chi-square test). For these neurons, the magnitude of response modulation was not significantly different between M593 and the control monkeys in either the PMv (Fig. S4A; self-action, *P* = 0.089; partner-action, *P* = 0.29; two-tailed Welch’s *t*-test) or MPFC (Fig. S4B; self-action, *P* = 0.34; partner-action, *P* = 0.28; two-tailed Welch’s *t*-test). In the PMv, the proportion of self-type neurons was greater in M593 than the control monkeys, with a marginally significant difference (M593 vs. M1486, *P* = 0.051; M593 vs. M1488, *P* = 0.098; chi-square test).

**Table 1:** Number of actor-related neurons in PMv.

	Self	Mirror	Partner	Total actor-related neurons	Total neurons sampled
M1486	52 (32.9)	62 (39.2)	44 (27.8)	158	348
M1488	40 (33.6)	43 (36.1)	36 (30.3)	119	217
M593	93 (42.9)	55 (25.3)^††,^*	69 (31.8)	217	447

Values in parentheses denote the percentage of total actor-related neurons.

^††^*P* < 0.01, M1486 vs. M593;

*P < 0.05, M1488 vs. M593; chi-square test.

**Table 2:** Number of actor-related neurons in MPFC.

	Self	Mirror	Partner	Total actor-related neurons	Total neurons sampled
M1486	29 (20.4)	44 (31.0)	69 (48.6)	142	308
M1488	18 (24.7)	24 (32.9)	31 (42.5)	73	172
M593	32 (28.3)	22 (19.5)^†,^*	59 (52.2)	113	237

Values in parentheses denote the percentage of total actor-related neurons.

^†^*P* < 0.05, M1486 vs. M593;

*P < 0.05, M1488 vs. M593; chi-square test.

The paucity of mirror-type neurons was previously reported in the MPFC of the autistic M344^12^. It has been hypothesized that mirror neurons might be dysfunctional in people with autism spectrum disorder^19–21^. In the human brain, invasive single-neuron recording is not feasible, except for medical diagnostic or therapeutic purposes. Instead, alterations in mirror neuron function have been explored using a *putative* electroencephalographic marker known as ‘mu suppression’. In humans, mu suppression is defined as the suppression of scalp potentials over the frontal cortex in the alpha band during action execution and observation^22,23^. In monkeys, similar suppression of high beta band activity has been consistently reported in the PMv^13,24–27^ and MPFC^13^.

**Figure 3:**
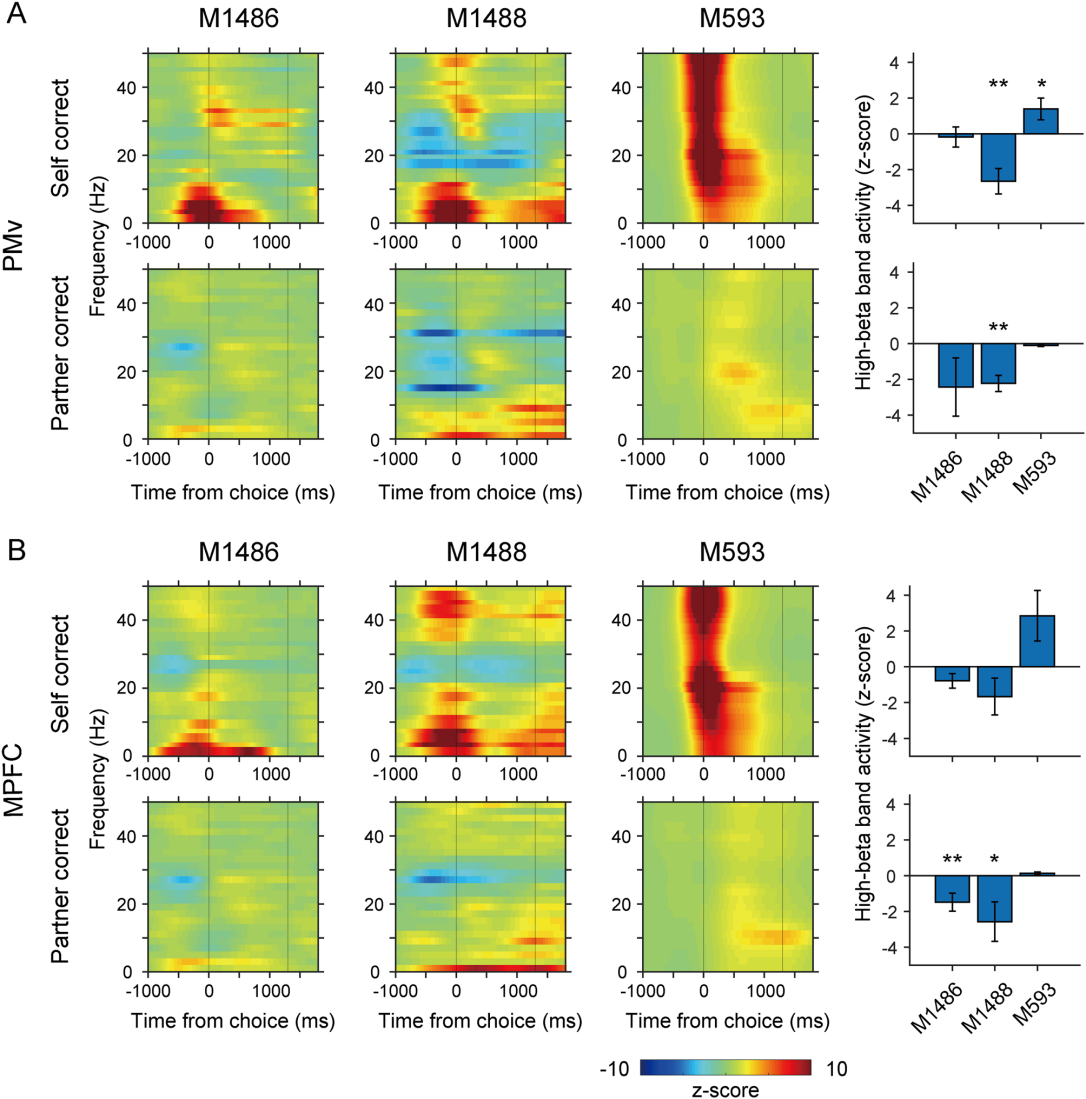
Lack of mu suppression in M593. (A) *Left*, LFP spectrograms recorded from the PMv for self-correct trials (top) and partner-correct trials (bottom). Vertical lines at 1300 ms indicate the time of reward feedback. *Right*, quantitative analyses of high-beta band activity (23–30 Hz). Asterisks indicate a significant difference from zero (*P < 0.05; **P < 0.01; Wilcoxon signed-rank test). (**B**) LFP spectrograms and high-beta band activity in the MPFC. Same conventions as in (**A**).

We found that mu suppression, measured using local field potentials (LFPs) at the high beta band (23–30 Hz), was completely absent in M593 in the PMv (Fig. 3A) and MPFC (Fig. 3B), in contrast to the control monkeys. In fact, the LFP power at the high beta band was mostly positive in M593. These findings support the prevailing view that mirror neuron activity at the single-neuron level and mu suppression at the LFP level are correlated. In addition to the difference in mu suppression, the LFP power at the gamma band was significantly greater in M593 than in the control monkeys during the self-actions (*P* < 0.05, two-tailed Welch’s *t*-test; Fig. S5).

### M639: Behavioral profiles

M639 (*M. fuscata*, male, 9 years old at the beginning of experiments) was enrolled in the study of the neural basis of social reward monitoring^14^. Behavioral abnormalities were not recognized in the home cage condition or during acclimatization to experimental environments. However, when a version of the Pavlovian conditioning procedure was introduced, we noticed that M639 was a slow learner, as described below. The purpose of this conditioning procedure was to examine whether the valuation of one’s own reward was affected by the reward of others^14^. For this, we placed M639 in a Pavlovian conditioning procedure while sitting face-to-face first with an object partner (i.e., a water-collecting bottle; ‘nonsocial condition’), followed by a monkey partner (‘social condition’).

**Figure 4:**
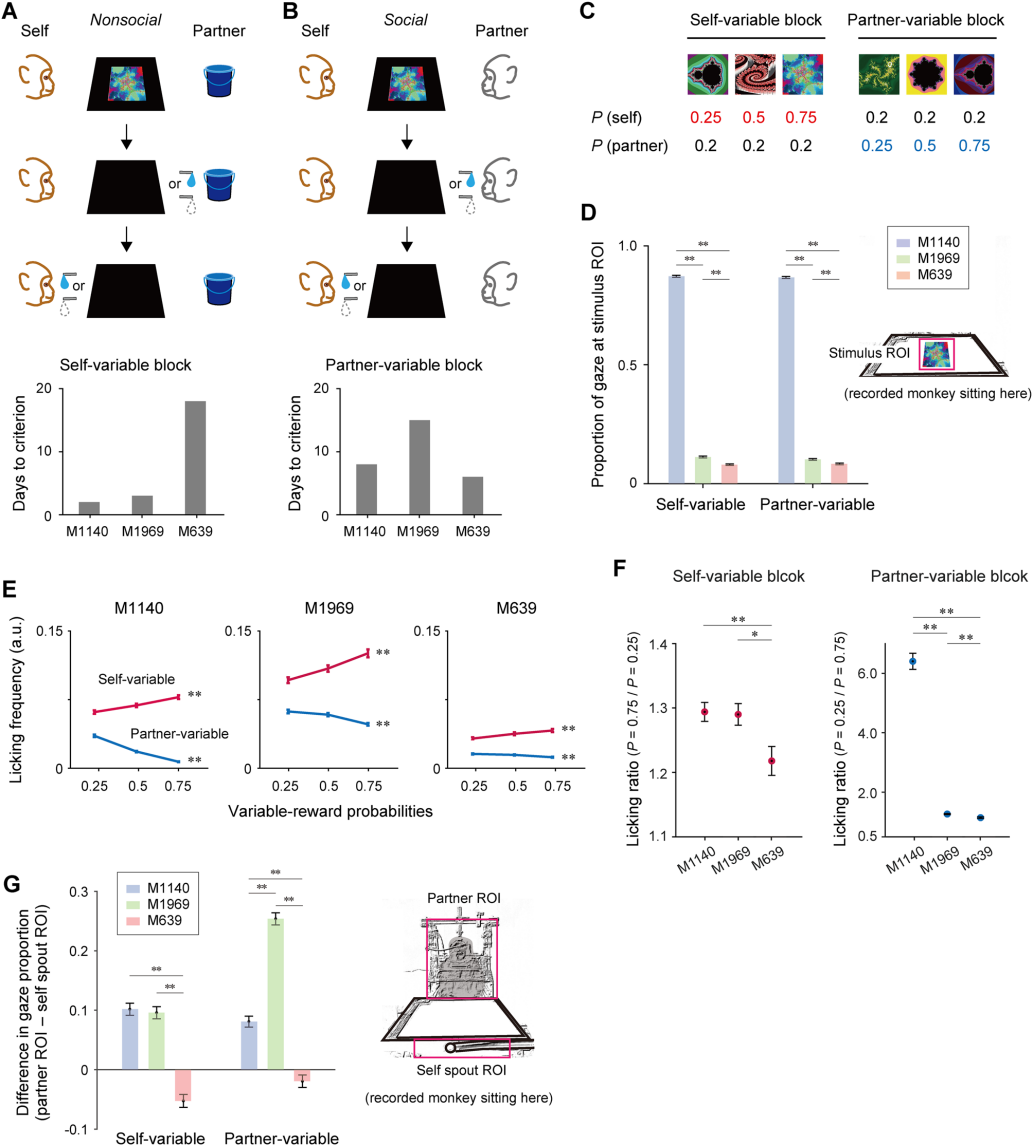
Pavlovian conditioning procedures for M639. (**A**) *Top*, sequence of events in non-social Pavlovian conditioning. *Bottom*, learning days required for the emergence of licking differentiation in the self-variable block. (**B**) *Top*, sequence of events in social Pavlovian conditioning. *Bottom*, learning days required for the emergence of licking differentiation in the partner-variable block. (**C**) Reward probability assigned to each conditioned stimulus. *P*(self), probability of self-reward. *P*(partner), probability of partner reward. (**D**) Proportions of gaze at the stimulus ROI during the stimulus period. Mean ± SEM; **P < 0.01, two-tailed Welch’s *t*-test. (**E**) Subjective value modulation expressed in anticipatory licking. Mean ± SEM; **P < 0.01, Spearman’s correlation test. Variable-reward probabilities denote reward probabilities that were variable in each block (corresponding to colored numerical values in (**C**)). (**F**) Licking ratio as a measure of the magnitude of subjective value modulation. Mean ± SEM; **P < 0.01, *P < 0.05, two-tailed Welch’s *t*-test. (**G**) Proportions of gaze at the partner ROI and self-spout ROI when the partner was rewarded (100–500 ms after reward was presented to the partner). Same conventions as in (**D**).

In the nonsocial condition, M639 was conditioned with several fractal stimuli for a liquid reward (Fig. 4A, top). Each trial started when a stimulus was presented at the center of the display. One second later, the stimulus presentation was discontinued and the reward feedback (delivery of a water reward or nothing) was presented first to the object partner and, one second later, to M639 (‘self’). The delivery of water reward to the object partner and M639 was accompanied by a low- and high-pitched tone, respectively.

We designed two blocks of trials that differed in reward contexts. In one block, called the self-variable block (Fig. 4C, left), the probability of reward for M639 was different depending on which of three stimuli was presented (*P* = 0.25, 0.5, or 0.75), whereas the probability of the object’s reward was invariable (*P* = 0.2). In the partner-variable block (Fig. 4C, right), the probability of the object’s reward was different depending on which of another three stimuli was presented (*P* = 0.25, 0.5 or 0.75), whereas the probability of the reward for M639 was invariable (*P* = 0.2). In both blocks, there was a constraint that M639 could be rewarded, albeit not always, when the object partner was not rewarded (exclusive reward recipients). The three stimuli in each block were presented in a pseudorandom order with the same overall frequency. The two blocks were alternately run after every 120 trials.

In the same project, we tested two other monkeys that were considered neurotypical (*M. fuscata*, males; M1140 and M1969) as controls. Their behavioral and neural data were reported previously^14^. We quantified the magnitude of licking movement during a stimulus presentation period as a measure of animals’ expectation of upcoming reward. In the control monkeys, the licking magnitude positively correlated with the probability of self-reward (Spearman’s correlation test, *P* < 0.01)^14^. This licking differentiation in the self-variable block emerged as early as the second (M1140) and third (M1969) training days (Fig. 4A, bottom). However, in M639, the emergence of licking differentiation was substantially delayed and appeared on the eighteenth training day (Fig. 4A, bottom). This learning delay might be associated, at least partly, with paying less attention to the reward-predictive stimuli in M639 than the control monkeys (Fig. 4D). In all monkeys, the licking magnitude was not differentiated in the partner-variable block in the nonsocial condition (Spearman’s correlation test, *P* > 0.01), suggesting that the reward to the physical object had no impact on the valuation of one’s own reward.

Once the monkeys had learned the basic structure of the conditioning procedure, we replaced the object partner with the monkey partner to create a social context (Fig. 4B, top). Consequently, all monkeys additionally exhibited licking differentiation in the partner-variable block, in such a way that the licking magnitude was negatively correlated with the probability of the partner reward (Fig. 4E). This finding suggests that the subjective value of one’s own reward decreased with increasing probability of other agents’ reward. At this stage, the three monkeys spent comparable amounts of time in developing the subjective value difference depending on the partner reward (Fig. 4B, bottom). However, the magnitude of the subjective value difference was significantly lower in M639 than M1140 and M1969 in both self-variable and partner-variable blocks (Fig. 4F). An additional test revealed that, when the partner monkey received a reward, the control monkeys looked longer at the partner than at one’s own spout region, whereas M639 did not show this partner-looking bias (Fig. 4G). These findings suggest that M639 was generally less sensitive to rewards and paid less attention to others, compared to the control monkeys.

### M639: Neuronal profiles

The brain MRI revealed no apparent structural abnormality in M639. To explore a possible neural correlate of behavioral properties of M639, we carried out multi-site, multi-electrode neural recordings from the MPFC and dopaminergic midbrain nuclei (DMN) while M639 was placed in the social Pavlovian conditioning procedure. Cortical recording sties were consistent between M639 and its controls (Fig. S6).

Consistent with our previous report on control monkeys^14^, four types of reward-related neurons were identified in the MPFC of M639: ‘self type’ that encodes the probability of self-rewards, ‘partner type’ that encodes the probability of partner-rewards, ‘mirror type’ that encodes both rewards, and ‘value type’ that encodes subjective reward value (“Methods” for the definition of neuronal classification). However, the proportion of reward-related neurons among all sampled neurons was significantly smaller in M639 than in the control monkeys in both early (151–450 ms from stimulus onset; Table S1; M639 vs. M1140, *P* = 3.4 × 10^–18^; M639 vs. M1969, *P* = 5.4 × 10^–6^; chi-square test) and late (701–1000 ms from stimulus onset; Table S2; M639 vs. M1140, *P* = 8.0 × 10^–22^; M639 vs. M1969, *P* = 4.6 × 10^–10^; chi-square test) epochs. The paucity of reward-related neurons was mainly associated with lower proportions of the self-type in the early epoch (Table S1) and all types in the late epoch (Table S2).

**Figure 5:**
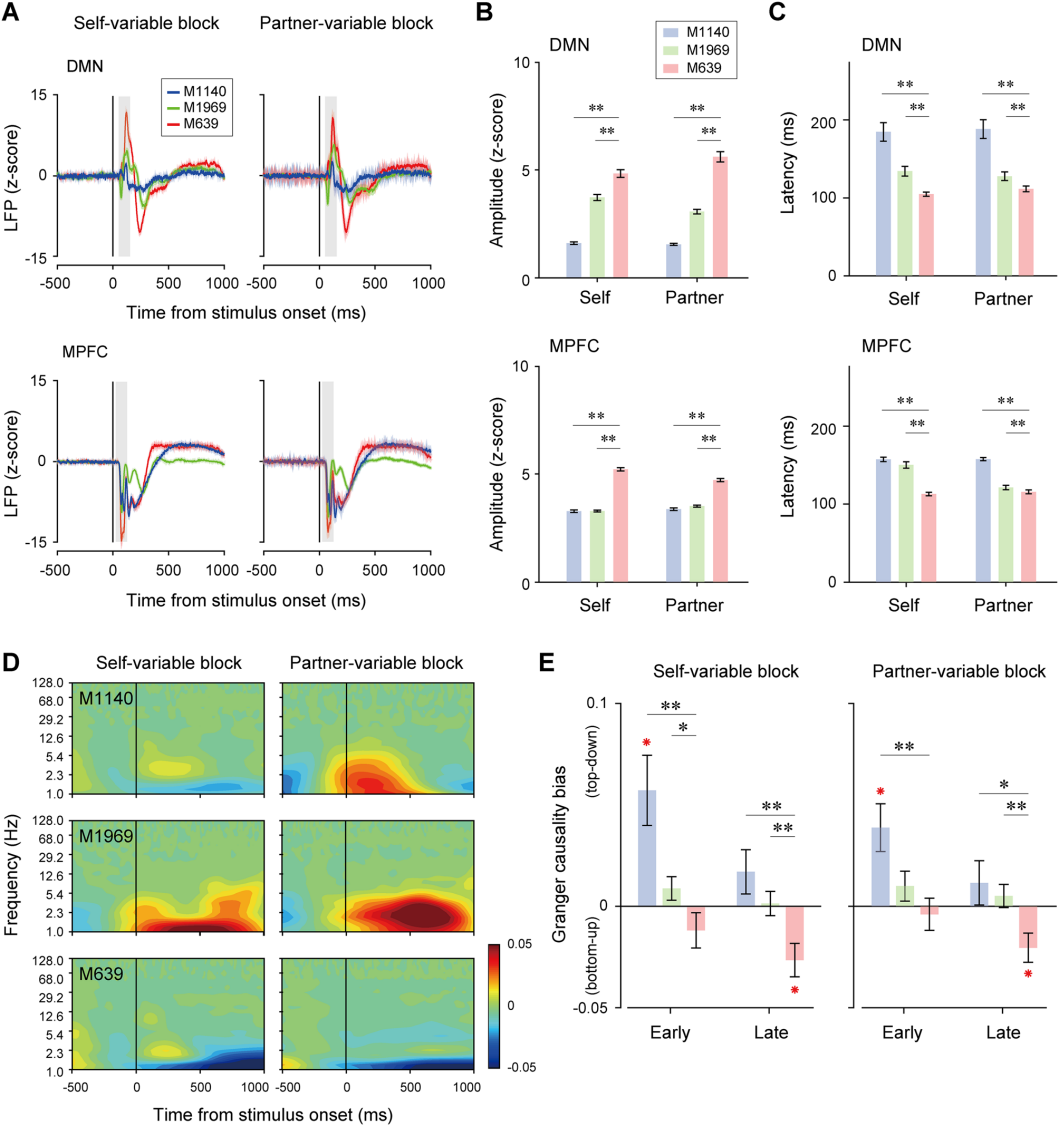
Neural activity properties in M639. (**A**) Responses of LFPs to conditioned stimuli. Gray rectangles indicate early LFP components. (**B**) Rectified amplitude of early LFP components. Mean ± SEM. Self, self-variable block. Partner, partner-variable block. **P < 0.01, two-tailed Welch’s *t*-test. (**C**) Latency of early LFP components. Same conventions as in (**B**). (**D**) Coherence between MPFC and DMN. (**E**) Causal information flow bias. Values indicate the proportion of channel pairs with significant Granger causality from the MPFC to DMN (top-down direction) *minus* the proportion of channel pairs with significant Granger causality from the DMN to MPFC (bottom-up direction). *P < 0.05, **P < 0.01, two-tailed Welch’s *t*-test. Red stars indicate a significant difference from zero (*P* < 0.01, two-tailed Wilcoxon signed-rank test). Other conventions are the same as in (**B**).

At the neural level, M639 also differed from its controls in visual responsiveness and inter-areal coordination. When reward-predictive visual stimuli were presented, the amplitude of LFP responses was markedly greater in M639 than the control monkeys in both the MPFC and DMN (Fig. 5A,B). In addition, the latency of the visual responses was consistently shorter in M639 than the control monkeys in both cortico-subcortical regions (Fig. 5C). In the DMN, single dopamine neurons also exhibited short-latency visual responses (Fig. S7). The matrix obtained from principal component analysis revealed that M639 occupied distant positions in the principal space (Fig. S3B), as was the case with M593.

In the control monkeys, field-field coherence was increased between the MPFC and DMN during the stimulus presentation period (Fig. 5D, top and middle). This increase was more evident at lower frequency bands (Fig. S8). However, in M639, the coherence increase was virtually absent (Fig. 5D, bottom). Furthermore, the direction of information flow between the two regions, as measured using Granger causality, was unique in M639. In the control monkeys, predominant information flow was in the top-down direction from the MPFC to DMN (Fig. 5E; positive values). In M639, the predominant flow was in the bottom-up direction from the DMN to MPFC (Fig. 5E; negative values).

### M593 and M639: Genetic profiles

M593 and M639 showed common behavioral phenotypes that were best described as learning delay and low levels of social performance monitoring. These phenotypic features were also observed in M344^12^. We therefore attempted to determine possible genetic correlates of the behavioral phenotypes common to the three monkeys. We first examined the possibility that there was any kinship ties between them. Large-scale genetic analysis using KING software^28^ estimated the kinship coefficient to be 0.0615 between M593 and M639, suggesting that these two monkeys were most likely cousins. The kinship coefficient between M593 and M344, and between M639 and M344 was − 0.0506 and 0.0029, respectively, suggesting that M344 had no apparent kinship with M593 or M639.

Next, we performed extensive genomic sequencing (exome) to search for genetic variants that were common to M593, M639, and M344, but absent in the control monkeys (M1486, M1488, M1140, and M1969). All seven monkeys used in this study were males. For this screening, we focused on loss-of-function or missense mutations reportedly linked to human brain disorders. We also focused on variants with frequencies of occurrence below 10% in the general macaque population (*n* = 1235; *M. fuscata*, *n* = 789; *Macaca fascicularis*, n = 326; *Macaca mulatta*, *n* = 120). Gene annotation was based on National Center for Biotechnology Information (NCBI; release 103) and Ensembl Gene Predictions (Ensembl; release 104).

**Figure 6:**
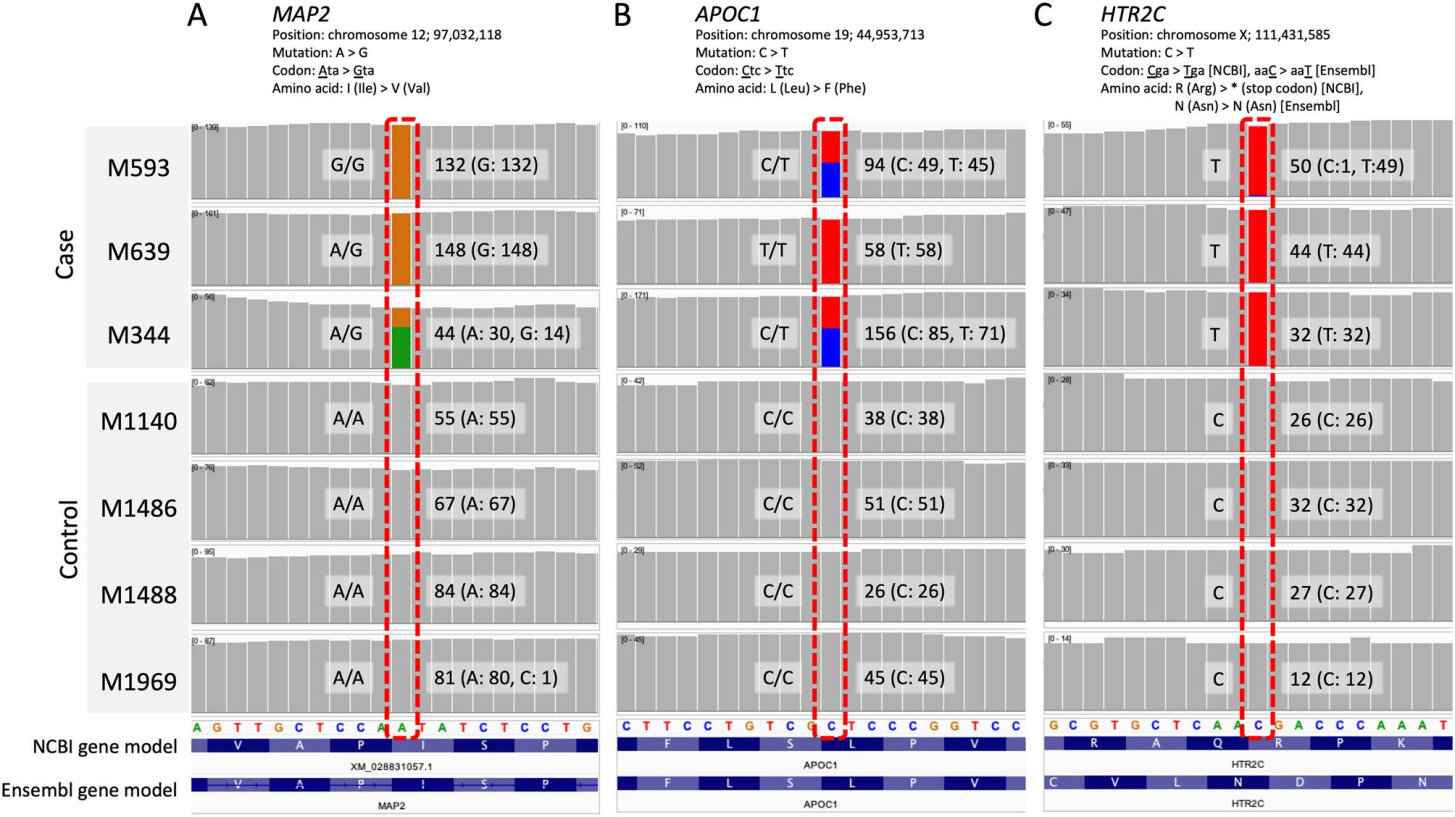
Genetic mutations specifically identified in monkeys with learning delay and low levels of social performance monitoring. (**A**) Exome sequence read coverage on *MAP2* in the case monkeys (M593, M639, and M344) and control monkeys (M1140, M1486, M1488, and M1969), showing adenine-to-guanine mutation. This missense mutation changed amino acids from isoleucine to valine in the three case individuals, not the control ones. Each mutation site’s genotypes and read depth (number of sequence reads) are depicted. (**B**) Read coverage on *APOC1* in the same seven monkeys. This mutation was also a missense mutation (amino acid changed from leucine to phenylalanine) specifically identified in the case monkeys. (**C**) Read coverage on *HTR2C* in the case and control monkeys. Depending on the gene annotation model used, the phenotypic effect of this mutation may vary; in the NCBI model, the mutation is a nonsense mutation and a stop codon appears. In comparison, in the Ensembl model, the mutation is a synonymous substitution and expected to have little effect on the phenotype. Note that *HTR2C* is located on chromosome X; therefore, the genotype in the seven male monkeys is monoallelic.

Three genetic variants satisfied the aforementioned conditions. The first variant was a missense mutation in *MAP2* (chromosome 12: 97,032,118; M593, homozygous; M639, homozygous; M344, heterozygous) (Fig. 6A). *MAP2* encodes microtubule-associated protein 2. The frequency of occurrence of this variant was 1.9% for homozygotes (*n* = 23/1235) and 5.9% for heterozygotes (*n* = 73/1235) in the macaque population. The second variant was a missense mutation in *APOC1* (chromosome 19: 44,953,713; M593, heterozygous; M639, homozygous; M344, heterozygous) (Fig. 6B). *APOC1* encodes apolipoprotein C1. The frequency of occurrence of this variant was 2.2% for homozygotes (*n* = 27/1235) and 14.7% for heterozygotes (*n* = 182/1235) in the population. The gene annotations for *MAP2* and *APOC1* were consistent between NCBI and Ensembl.

The third variant was a nonsense mutation in *HTR2C* (chromosome X: 111,431,585; hemizygous for all target monkeys) (Fig. 6C). *HTR2C* is located on the X chromosome and encodes 5-hydroxytryptamine (serotonin) receptor 2C. The frequency of occurrence of this variant was 1.9% for hemizygotes (*n* = 8/411) in the male population. This variant, which is present in the so-called truncated isoform, has been reported in M344 on the basis of an earlier version of Ensembl (release 78)^12^. Although annotations based on NCBI still predict that this variant causes a nonsense mutation, it is considered a silent mutation according to the latest version of Ensembl (release 104). Other loss-of-function mutations found individually in M593, M639, and M344 are shown in Table S3.

## Discussion

We demonstrated the neural and genetic correlates of learning delay and low levels of social performance monitoring in two Japanese macaques (M593 and M639) under laboratory conditions. Apart from slower habituation to the experimental environment, M593 showed obsessions with rules, as reflected in prolonged perseveration errors, and choice strategies suggestive of maladaptive social action monitoring. The same pattern of choice strategies was reported in the autistic monkey M344^12^, as well as in one (M1488) of the control monkeys after selective blockade of the PMv-to-MPFC pathway^13^. In M639, we observed a marked delay in the acquisition of classical conditioning, generally low sensitivity to reward probabilities, and indifference to other’s outcomes. Exome analyses revealed that M593 and M639 shared genetic similarity, suggesting that they were most likely cousins. Moreover, M593, M639, and previously reported M344 had rare coding variants in *MAP2*, *APOC1*, and *HTR2C*. Although the latest annotation for *HTR2C* is not consistent between NCBI (release 103) and Ensembl (release 104), and thus the etiological relevance of this gene to the observed phenotypes cannot be determined unequivocally, our findings suggest the possibility that *MAP2*, *APOC1*, and potentially *HTR2C* are linked to the phenotypic expression of slow learning, maladaptive performance monitoring of others, obsessions with systems, and egocentric attentional priority, as often seen in neurodevelopmental disorders.

MAP2 encodes a neuron-specific cytoskeletal protein, which plays a role in dendritic arborization during development^29^. The possibility that *MAP2* is involved in neuropsychiatric illnesses has been described in children with 2q34 deletion who exhibited autistic and Rett-like features^30,31^ or developmental delay, epilepsy, and problems in social distance regulation and impulse control^32^. Expression levels of MAP2 are markedly reduced in the frontal cortex in autistic adult individuals^33^. MAP2 is differentially phosphorylated in the primary auditory cortex in schizophrenic individuals, which is thought to reduce the binding of this protein to microtubules^34^. Another line of evidence indicates that MAP2 is involved in the induction of long-term potentiation in the mouse hippocampus^35^, pointing to the role of MAP2 in learning and memory.

*APOC1* encodes apolipoprotein C1, which is detected in astrocytes in the central nervous system^36^. *APOC1* polymorphisms have been associated with increased risk of Alzheimer’s disease^37,38^ and age-associated memory impairment^39^. The expression levels of *APOC1* are reduced in the frontal cortex of Alzheimer’s disease patients^36^.

*HTR2C* encodes a dominant subtype of the serotonin receptor in the central nervous system. The receptor subtype 2C is preferentially expressed in the striatum, hippocampus, hypothalamus, substantia nigra, amygdala, and neocortical areas^40–43^. According to the NCBI annotation, the mutation identified in M593 and M639 leads to the elimination of the last 70 amino acids of the truncated isoform^12^. This isoform is the primary transcript in the macaque brain during early developmental periods^12^. The serotonin 2C receptor is associated with various neuropsychiatric disorders, such as eating disorder, seizures^44^, Prader-Willi syndrome^45^, depression, bipolar disorder^46–48^, and autistic behavior^49^.

M593 shared behavioral features with M344, which is considered a naturally occurring model of autism spectrum disorder^12^. These features included inflexible adherence to routines and maladaptive monitoring of others’ action. Our notable finding was that the proportion of mirror-type neurons in the PMv and MPFC was significantly lower in M593 than the control monkeys. Mirror neurons in the mirror system have been hypothesized to be dysfunctional in people with autism spectrum disorder^19–21^. A number of findings in human neuroimaging and electroencephalographic studies are consistent with this “broken mirror hypothesis”^22,50–52^. However, given the fact that mirror neurons can be defined only at the single-neuron level, there has been no direct evidence for the broken mirror hypothesis. In this light, the present study provides the first evidence for deficient organization of mirror neurons in the PMv, a critical node in the mirror system.

Instead of recording directly from mirror neurons, human studies have relied on the phenomenon called mu suppression as a noninvasive, electrophysiological marker of mirror neuron activity. Mu suppression in humans is characterized as suppression of scalp potentials in the alpha band during action execution and observation; this suppression is absent in people with autism spectrum disorder^22,23^. However, associations between mirror neuron activity at the single-neuron level and mu suppression at the field potential level are speculative. In this study, we showed that mu suppression is absent in the monkey PMv and MPFC, which had proportionately fewer mirror neurons than the control monkeys. Our findings lend support to the prevailing hypothesis that mirror neuron dysfunction is a neurobiological marker of the autistic phenotype, and the absence of mu suppression is an electrophysiological marker of mirror neuron dysfunction.

In addition to the lack of mu suppression, there was another difference in LFP spectrograms between M593 and its control monkeys during self-actions. In the control monkeys, the increase in LFP power was predominant in the delta, theta, and alpha bands. In M593, LFP power increased diffusely including the gamma frequency band. Although functional significance of this difference remains to be determined, one possibility is that prominent gamma-band power might be associated with heightened attentional priority to the self-actions, given that gamma-band activity is associated with attentional processes^53,54^ and increased gamma-band activity can be observed in the autistic brain^55,56^.

In M639, we observed enhanced visual responsiveness in both cortical (MPFC) and subcortical (DMN) regions. We also observed the predominance of information flow in the bottom-up direction (i.e., DMN to MPFC), as opposed to the top-down direction that is observed in the control monkeys^14^. The functional significance of these neural phenotypes is not immediately clear. However, we speculate that the former might be associated with imbalance between excitatory and inhibitory neural signals. It has been postulated that some forms of autism are caused by a disproportionately high level of excitation in sensory, mnemonic, social, and emotional systems^57^. Such excitatory bias might lead to hypersensitivity to sensory input and impulsivity, as typically seen in people with autism spectrum disorder and attention deficit hyperactivity disorder, respectively^58^. The latter finding, i.e., bottom-up information flow bias, might render the animal indifferent to others, considering that the MPFC is a critical node involved in moderating the self and others^17^.

In M639, the proportion of reward-related neurons was significantly smaller than the control monkeys. Unlike M593, in which mirror-type neurons were smaller in proportion compared to its controls, the relative paucity of task-related neurons in M639 was not cell-type specific. This finding suggests that the functional role of ‘mirror-type’ neurons is different between action and reward domains. It is conceivable that action-related mirror-type neurons respond to ongoing visible movements of the self and other, while reward-related mirror-type neurons respond to the likelihood of upcoming reward to the self and other. The likelihood of events per se is not directly observable and only acquired through repetitive experiences. In this light, mirror-type neurons in the reward domain, at least in our task context, might encode more abstract-level of information.

We should discuss two issues regarding the limitations of interpretation owing to experimental design. The first concerns the small number of animals involved. In the present study, only three monkeys—one case monkey and two control monkeys—underwent behavioral and electrophysiological testing in each task condition. Although differences in behavioral and neural properties were reported on the basis of statistical significance between the case and control monkeys, it is also true that such properties were often variable and statistically different between the control monkeys. This variability made it difficult to evaluate whether the case monkeys deviated from the distribution of control monkeys and could be described as “unique.” Second, the two case monkeys were examined using different behavioral tasks, i.e., M593 in operant conditioning and M639 in classical conditioning. In the case of the phenotype-driven approach, however, researchers do not know in advance which monkeys would exhibit potentially deviant behavioral phenotypes. Rather, it is usually the case that monkeys are blindly assigned to different experimental projects on the assumption that they are neurotypical. For this reason, detailed advance planning is technically demanding in phenotype-driven cognitive genomics. In this light, the accumulation of monkeys with similar phenotypes, along with a set of neural and genetic profiles, is of crucial importance, just like clinical case reports in humans. Despite differences in task contexts, we found common behavioral phenotypes between the case monkeys. This point is important, because in autism spectrum disorders, deficits in social communication and social interaction are not confined to a particular context but are observed persistently across multiple contexts^58^.

In summary, we demonstrated that the two macaque monkeys exhibited learning delay and low levels of social performance monitoring. Animals with potentially pathological cognitive-behavioral phenotypes are usually not considered suitable for use in basic research. Yet, data from such animals can provide important insights, similar to human clinical case reports, into a triadic relationship between genes, brain, and cognition, as has been advocated by previous work, including ours^12,59,60^. The phenotype-driven cognitive genomics strategy used in the present study is useful to explore novel candidate genes responsible for particular cognitive phenotypes, because it does not require pre-selection of target genes. We expect that this approach will contribute to a better understanding of the genetic and neurobiological mechanisms underlying neuropsychiatric disorders and the development of nonhuman primate models of those disorders.

## Methods

### Subjects

For the first experiment on the neural basis of social action monitoring, three male monkeys were used [*M. fuscata*; M593 (aged 5 years, 6.2 kg), M1486 (aged 6 years, 5.1 kg), and M1488 (aged 6 years, 5.0 kg)]. For the second experiment of the neural basis of social reward monitoring, four male monkeys were used [*M. fuscata*; M639 (aged 12 years), M1140 (aged 7 years), M1969 (aged 5 years), and D (aged 12 years)]. All monkeys, except for one (D), underwent neural recordings. These monkeys were housed in individual cages, but were capable of communicating with each other both visually and verbally. The animal care and experimentation protocols were approved by the Institutional Animal Care and Use Committee of National Institutes of Natural Sciences, and were carried out in accordance with the guidelines described in the US *National Institutes of Health Guide for the Care and Use of Laboratory Animals*. This study is reported in accordance with ARRIVE guidelines.

### Behavioral procedures

#### Visually guided reaching task (M593, M1486, and M1488)

A square panel was placed horizontally on the front of a primate chair (Fig. 1A). Four buttons were mounted on the panel: a circular one on the near side as a start button and three rectangular ones on the far side as target buttons. Each trial started when a start button was illuminated. The monkeys were required to hold the start button for 0.7–1.3 s. One of the three target buttons was illuminated as a cue to make a reach. The subject was rewarded with a drop of water 1.3 s after the target button was pressed, if the reaction time was less than 3 s. A high-pitched tone (1 kHz) was delivered as feedback for each button press. The position of the correct target remained the same for a block of 11–17 trials.

#### Reversal learning task (M593, M1486, and M1488)

The monkeys were initially trained to individually perform a reversal learning task (nonsocial reversal learning task; Fig. 1B)*. *The sequence of events was the same as the visually-guided reaching task, but the targets were illuminated after the subject successfully held the start button. Only one of the three buttons was the correct target and its position changed after every 11–17 trials with no prior notice. The monkeys were rewarded with a drop of water 1.3 s after the correct target was pressed. Then, the monkeys were trained to perform the reversal learning task with another monkey (social reversal learning task; Fig. 2A). For this task, M1 (recorded monkey or self) and M2 (partner) faced each other with their chair panels positioned near one another (distance =  ~ 1 cm). M1 and M2 were assigned different roles: actor and observer. The sequence of events in each trial was essentially the same as in the nonsocial reversal learning task, but only the target buttons on the actor’s side were illuminated. The observer was required to hold the start button throughout the trial. The two roles alternated after every three trials. When the actor’s choice was correct, both subjects were rewarded. Neither subject was rewarded when the actor’s choice was incorrect. During neuronal data collection, M593 was paired with M1488. In some later sessions, M593 was paired with a human experimenter, as M1488 was engaged in another experiment. Note that the proportion of mirror-type neurons in M593 was not significantly different between the monkey and human partners (PMv, *P* = 0.14; MPFC, *P* = 0.68; chi-square test).

#### Pavlovian conditioning procedures (M639, M1140, M1969, and D)

The monkeys M639, M1140, and M1969 (collectively designated as M1) were conditioned with fractal visual stimuli for a liquid reward. The monkeys initially faced an object partner (a water collecting bottle) as a nonsocial condition (Fig. 4A) and, several days later, faced a monkey partner (monkey D) as a social condition (Fig. 4B). The two types of partners were collectively designated as M2. The temporal sequence of events was the same between the two conditions except for the difference in animacy of the partner.

Each trial began with presentation of a visual fractal stimulus (188 × 202 mm) at the center of a monitor. After 1 s, the stimulus disappeared and a trial outcome (delivery or non-delivery of water reward) was presented first to M2 and, 1 s later, to M1. The outcome was determined on the basis of reward probabilities associated with each stimulus (see below). The delivery of a reward to M2 and M1 was accompanied by a low-pitched (125 Hz) and high-pitched (1 kHz) tone, respectively.

Two trial blocks were alternately run after every 120 trials: M1/self-variable and M2/partner-variable blocks. In the M1-variable block, three different stimuli were used; each stimulus was associated with M1-reward at different probabilities (*P* = 0.2, 0.5, and 0.75), while the three stimuli were associated with the same M2-reward probability (*P* = 0.2). In the M2-variable block, another three stimuli were used; each stimulus was associated with M2-reward at different probabilities (*P* = 0.2, 0.5, and 0.75), while the three stimuli were associated with the same M1-reward probability (*P* = 0.2). The monkeys obtained the same total amount of rewards in the two blocks. In either block, both animals were never rewarded on the same single trial. Therefore, M1 had an opportunity to receive a reward only when M2 had not been rewarded. This indicates that the final outcome in each trial was M1-rewarded, M2-rewarded, or neither-rewarded.

### Surgical procedures

The monkeys were anesthetized with intramuscular injections of ketamine HCl (10 mg/kg) and xylazine (1–2 mg/kg), or medetomidine (0.05 mg/kg) and midazolam (0.25 mg/kg). The general anesthetic state was maintained with isoflurane (0.8–2%). After exposing the skull, acrylic screws were installed to fasten dental acrylic head implants to the skull under aseptic surgical conditions. A nonmetal head holder and recording chambers were positioned stereotaxically and secured with dental acrylic. Craniotomy was performed after the monkeys had been trained on the behavioral procedures described previously. Antibiotics and analgesics were administered after the surgery.

### Behavioral recording procedures

Stimulus presentation, behavioral data collection, and reward delivery were controlled by a personal computer running MATLAB (MathWorks Inc., Natick, MA, USA) with the MonkeyLogic toolbox^61^. The water reward was delivered through a spout under the control of a solenoid valve placed outside a sound-attenuated room. Licking movements were sampled at 1 kHz, filtered (100–200 kHz), and amplified with a vibration sensor attached to the reward spout (AE-9922; NF Corporation). Eye position was monitored using an infrared video tracking system at a time resolution of 500 Hz and spatial resolution of 0.1° (iRecHS2, Human Informatics Research Institute, National Institute of Advanced Industrial Science and Technology). The monkeys’ overt movements were continuously monitored using a custom-made video-capturing system on MATLAB.

### Neural recording procedures

For electrophysiological experiments, single-unit activity and LFPs were recorded using multi-contact electrodes (U- or S-probe, Plexon Inc., Dallas, TX, USA). These electrodes consisted of 16 channels, which were arranged linearly with 200 μm spacing and impedance of 0.3–0.5 MΩ at 1 kHz. For single-unit recordings, signals were amplified and bandpass-filtered (150 Hz to 8 kHz; OmniPlex system; Plexon Inc.), and then the activity of each unit was isolated online using a template-matching spike discriminator (SortClient; Plexon Inc.) or offline on the basis of waveform features (Offline Sorter, Plexon Inc.). Clusters that were not clearly separated from noise were excluded. In isolated clusters, presumed single units with inter-spike intervals less than 2 ms were excluded. In two-dimensional feature space of the principal components, values with Mahalanobis distance greater than ± 3 SD (standard deviation) from the centroid of the cluster along the principal axis were classified as outliers and excluded. For LFP recordings, signals were bandpass-filtered (0.2–500 Hz) and digitized at 1 kHz (OmniPlex system; Plexon Inc.). In each session, an oil-driven micromanipulator (MO-97A or MO-971A; Narishige, Tokyo, Japan) was used to advance the probe through a stainless steel guide tube that was held in place by a grid. The grid allowed recording penetrations with a spatial resolution of 0.5 mm.

### Identification of recording sites

#### PMv

We initially mapped the frontal eye field (FEF) by exploring the rostral bank of the arcuate sulcus using intracortical microstimulation (ICMS; cathodal pulses of 0.2 ms duration at 333 Hz, 11 or 44 pulses). The FEF was identified by saccadic eye movements evoked by ICMS with low thresholds^62^ (typically 11 pulses with < 50 μA current intensity). During the mapping, a few penetrations showed no neuronal activity immediately posterior to the FEF, the coordination of which was noted as the spur of the arcuate sulcus. The region immediately posterior and ventral to the arcuate spur was defined as the PMv. Distal movements evoked by ICMS were also confirmed in this region. We also ‘clinically’ examined the response properties of neurons encountered during physiological mapping^63,64^. For example, neuronal responses were monitored when the monkeys or experimenter made a grasping action toward a food item to test mirror properties.

#### MPFC

The MPFC contains the prefrontal area 9 and its caudally adjacent pre-supplementary motor area (pre-SMA). The pre-SMA was characterized by complex movements involving multiple joints following ICMS (44 pulses) and preferential responses to visual stimuli over somatosensory stimuli^65^. The most rostral portion of the recording site was 12 mm anterior to the physiological border between pre-SMA and SMA.

#### DMN

Recordings from the DMN were mainly from the substantia nigra pars compacta (SNc) and ventral tegmental area (VTA). To identify these regions, we used the substantia nigra pars reticulata (SNr) and third cranial nerve as landmarks. SNr neurons exhibited a high-frequency spontaneous discharge, which was often inhibited by visual stimulation or saccadic eye movement^66^. The third cranial nerve showed regular and tonic firing of noticeably high frequency that was closely associated with eye position. Presumed dopamine neurons in the SNc and VTA were identified on the basis of their firing properties: irregular firing with low spontaneous discharge rates (~ 5 Hz), broad spike potentials, and phasic excitation in response to unexpected rewards. The recording site was confirmed by histological examinations^14^.

### Statistical analysis

Although statistical methods were not used to predetermine sample sizes, our sample sizes were similar to those used in previous studies^13,14^. All well-isolated neurons were included in the neuronal recordings to prevent sampling bias. Blinding was not performed for investigators involved in data collection and analysis. No data were excluded unless otherwise stated. All statistical procedures were assessed by two-tailed tests and carried out using MATLAB Statistics and Machine Learning Toolbox, Signal Processing Toolbox, Parallel Computing Toolbox, Control System Toolbox, and Multivariate Granger Causality Toolbox (version 2018b and 2020b; MathWorks Inc.).

### Data analysis

#### Performance in the nonsocial reversal learning task

The progress in task learning was evaluated using a scoring system (Fig. 1C). Specifically, a score of − 1 was assigned to a trial in which the monkeys selected the correct target in the preceding block (i.e., incorrect target in the current block), 0 was assigned to the choice of the correct target in the current block, and + 1 was assigned to the choice of the remaining target that was not correct in either the preceding or current block. Because of the unpredictable nature of block switches, a score of − 1 was the typical result in the first trial in each block (i.e., switch trial). Well-trained monkeys were expected to change their choice in the next trial, which resulted in scores of 0 or 1, with equal frequencies. Therefore, the score in the second trial averaged across the blocks should be positive if optimal target switching dominates, whereas the score should be negative if perseverative errors dominate.

#### Trial selection for evaluation of M1’s performance following M2’s choice error in social reversal learning task

To evaluate M1’s performance following M2’s error in non-switch trials (‘choice error’ case), we selected M1-actor trials that met the following two conditions: (1) the M1-actor trial was immediately preceded by M2’s choice error and (2) the correct target in the current block had been selected by the either monkey before M2 made a choice error. Note that M1’s optimal performance in this situation was to continually choose the correct target in the current block.

#### Trial selection for evaluation of M1’s performance following M2’s switch error in social reversal learning task

To evaluate M1’s performance following M2’s error in switch trials (‘switch error’ case), we selected M1-actor trials that met the following two conditions: (1) the M1-actor trial was immediately preceded by M2’s switch error in the first trial of the current block and (2) in the switch trial, M2 selected the correct target in the preceding block. Note that M1’s optimal performance in this situation was to choose one of the two targets that were not chosen by M2 in the switch trial.

#### Definition of actor-related neurons in the social reversal learning task

Neuronal activities were quantified in a control period (0–600 ms before target onset) and a peri-action period (from 400 ms before to 200 ms after target button press). Then, a series of analyses was performed to classify individual neurons into self, mirror, and partner types (Tables 1 and 2) as defined previously^13^, as follows. First, the effects of the two factors [agent (self or partner) and performance outcome (correct or incorrect)] in the peri-action period were examined by a two-way analysis of variance (*P* < 0.05). Neurons with a significant main effect of agent were judged to be agent-selective (self or partner type). Neurons were defined as self type if their activities in the peri-action period were significantly higher (excitatory) or lower (inhibitory) in self-action trials than partner-action trials (*P* < 0.05, Tukey–Kramer post-hoc test), and their activities in the peri-action period were significantly higher (excitatory) or lower (inhibitory) than those in the control period (*P* < 0.05, paired *t*-test). Neurons were defined as partner type if their activities in the peri-action period were significantly higher (excitatory) or lower (inhibitory) in partner-action trials than self-action trials (*P* < 0.05, Tukey–Kramer post-hoc test), and their activities in the peri-action period were significantly higher (excitatory) or lower (inhibitory) than those in the control period (*P* < 0.05, paired *t*-test). Finally, neurons with no significant main effect of agent were classified as mirror type, if their activities in the peri-action period were significantly higher (excitatory) or lower (inhibitory) than those in the control period (*P* < 0.05, paired *t*-test) in both self-action and partner-action trials.

To compare the response magnitude of mirror-type neurons between M593 and control monkeys (M1486 and M1488), we computed the absolute value of differences in firing rates between the control period and peri-action period for each neuron (Fig. S4). The significance of difference was tested using two-tailed Welch’s *t*-test (*P* < 0.05) separately for the correct self-action and partner-action trials.

#### Definition of reward-related neurons in the social Pavlovian conditioning procedure

Neuronal activities were quantified during an early (151–450 ms from stimulus onset) and late (701–1000 ms from stimulus onset) epochs in the stimulus period. The significance of associations between activity and variable reward probability was assessed with linear regression separately in the M1-variable and M2-variable blocks, and fitted slopes and intercepts were obtained for each neuron. On the basis of the significance of the slope coefficient (*P* < 0.01), each neuron was classified into one of the four types as reported previously^14^: self, partner, mirror, and value (Tables S1 and S2). The self-type neurons were defined as those exhibiting a significant slope coefficient (either positive or negative) only in the M1-variable block. The partner-type neurons were defined as those exhibiting a significant slope coefficient (either positive or negative) only in the M2-variable block. The mirror-type neurons were those exhibiting a significant slope coefficient in both M1-variable and M2-variable blocks in the same direction (i.e., both positive or both negative). The value-type neurons were those exhibiting a significant slope coefficient in both M1-variable and M2-variable blocks in the opposite direction.

#### Gaze analysis

M1’s gaze positions within a specified region of interest (ROI) were quantified. In the social reversal learning task, the proportion of M1’s gaze at the M2’s correct target (M2-target ROI) was quantified during a period 400–200 ms before M2’s target press (Fig. 2D). In the social Pavlovian conditioning procedure, the proportion of M1’s gaze at the visual stimulus on the monitor (stimulus ROI) was quantified during early (151–450 ms) and late (701–1000 ms) epochs in the stimulus presentation period (Fig. 4D). In addition, the proportions of M1’s gaze at M2 (partner ROI) and M1’s spout region (self spout ROI) were quantified in M2-rewarded trials during 100–500 ms after reward onset (Fig. 4G).

#### Definition of licking differentiation in Pavlovian conditioning procedures

When the magnitude of licking movement was significantly correlated with variable reward probabilities in either the M1-variable or M2-variable block for two successive days (*P* < 0.01, Spearman’s correlation test), the first day was defined as the day of licking differentiation in the corresponding block (Fig. 4A,B). This analysis was performed for a period of 401–700 ms and 701–1000 ms after stimulus onset in the M1-variable and M2-variable blocks, respectively.

#### Licking ratios

The magnitude of subjective value modulation during the stimulus presentation period was quantified by computing a licking ratio. Licking ratios in the self-variable block were defined as licking responses in the highest-valued trials (i.e., self-reward probability = 0.75) divided by those in the lowest-valued trials (self-reward probability = 0.25). Licking ratios in the partner-variable block were defined as licking responses in the highest-valued trials (partner-reward probability = 0.25) divided by those in the lowest-valued trials (partner-reward probability = 0.75). For this computation, only non-zero data values were used. Outliers were defined as data values exceeding 3 SD from the mean and were excluded from the analysis.

#### Time–frequency domain LFP analysis in the social reversal learning task

For constructing spectrograms of LFPs in the PMv and MPFC, power in each frequency band was computed in 1-ms and 1-Hz steps from 1 to 50 Hz (Fig. 3). The resultant spectrograms were z-score normalized per frequency using the activity 0–500 ms before target onset and averaged across sessions. The strength of activity (23–30 Hz) in the high-beta band was quantified by averaging the z-scored spectrogram 0–600 ms before the target button was pressed, which was then subjected to two-tailed Student’s *t*-test (*P* < 0.05) to determine the significance of difference from zero. The strength of activity in the gamma band (31–55 Hz) was also quantified in the same way and compared between the monkeys (two-tailed Welch’s *t*-test).

#### Latency and amplitude of neural responses in the social reversal learning task

To quantitatively compare the latency and amplitude of LFP responses between the monkeys, raw LFP signals were averaged across channels and trials for the correct self-action and partner-action, and then normalized with baseline activity (0–500 ms before target button onset) using a z-score normalization procedure. The amplitude was computed using the average of absolute z-score values during 31–180 and 31–150 ms after target button onset for the PMv and MPFC, respectively. The latency was defined as the first bin at which the z-score values exceeded 1.5 SD for at least 3 consecutive bins (1-ms resolution). Data values recorded within 30 ms after target button onset were excluded.

#### Latency and amplitude of neural responses in the social Pavlovian conditioning procedure

To assess the latency and amplitude of LFP responses, raw LFP signals were averaged across contacts and trials, and then normalized with baseline activity (0–500 ms before stimulus onset) using a z-score normalization procedure (Fig. 5A–C). The amplitude was computed using the integral of absolute z-score values during 51–150 and 26–125 ms after stimulus onset for the DMN and MPFC, respectively. The latency was defined as the first bin at which the z-score values exceeded 3 SD for at least 30 consecutive bins (1-ms resolution). Data values recorded within 51 and 26 ms after stimulus onset from DMN and MPFC, respectively, were excluded. For presumed single dopamine neurons, latency was defined as the bin with the peak amplitude (1-ms resolution).

#### Principal component analysis (PCA) of LFPs

To visualize differences in neural activities between the monkeys in the social reversal learning task, we applied PCA to matrix data of the amplitude and latency of LFP responses in the PMv and MPFC. A matrix of the amplitude and latency in correct self-action trials and correct partner-action trials (columns) was obtained per contact (rows), and the matrices across sessions and subjects were concatenated along the rows. The resultant matrix was then fed to PCA, and the first two principal components were used to inspect the distribution differences across the monkeys. The same procedures were applied to LFP responses in the DMN and MPFC during the social Pavlovian conditioning procedure; here, the matrix columns were constructed with the amplitude and latency in the M1-variable and M2-variable blocks. These matrix data in each brain region were separately constructed and analyzed. For the PMv and MPFC, LFP signals in all contacts were used. For the DMN, LFP signals in contacts at which unit activity of presumed dopamine neurons was recorded were used.

#### Field-field coherence

The first derivative of the LFPs from adjacent contacts was computed per electrode in the superficial direction to generate 15 bipolar LFPs. This procedure reduced potential artifacts and spurious correlations between the electrode channels, resulting in more spatially precise evaluation of signal interaction. Bipolar LFPs of all single trials around stimulus onset (5 s, − 2.0 to + 3.0 s from stimulus onset) were concatenated to generate one long time series convolved with a complex Morlet wavelet function and divided into the original 5-s LFP segments. Coherence was calculated for the LFP pairs between MPFC and DMN (1–128 Hz in a logarithmic step, *n* = 24). Each coherence was normalized by subtracting the baseline coherence signals (0–500 ms before stimulus onset) from the stimulus-period coherence signals (Fig. 5D). To quantify the coherence strengths in delta-to-high gamma frequency bands (δ, 1–3 Hz; θ, 4–7 Hz; α, 8–12 Hz; low β, 13–20 Hz; high β, 21–30 Hz; low γ, 31–49 Hz; high γ, 50–128 Hz), the averaged values were used in each band (Fig. S8).

#### Granger causality

In an attempt to evaluate information flow between MPFC and DMN, Granger causality (GC) analysis^67^ using a multivariate linear vector autoregressive (MVAR) model^68^ was applied to the bipolar LFPs simultaneously recorded from the two regions (Fig. 5E). The bipolar LFP segments in the early and late stimulus epochs were analyzed separately in each block. Akaike information criteria were used to estimate the best model order up to 50 ms. The MVAR model parameters for the selected model order were estimated using ordinary least squares regression. The autocovariance sequence from the MVAR parameters was calculated for the LFP time series data without the problems of collinearity, non-stationarity, or heteroscedasticity^67,68^. Data with such problems were excluded. Finally, the time-domain pairwise conditional GC was estimated using F-testing with false discovery rate (*Q* < 0.05). The numbers of channel pairs with significant GC for the MPFC-to-DMN direction and DMN-to-MPFC direction were counted for quantitative comparisons.

#### Exome analysis

Exon sequencing (exome) targeting 503 human neuropsychiatric disease-related genes was performed in 1235 macaques, including M593, M639, M1486, M1488, M1140, and M1969 (*M. fuscata*, *n* = 789; *M. fascicularis*, *n* = 326; *M. mulatta*, *n* = 120). We trimmed adapter sequences and low-quality bases, and mapped them to rheMac10 using bwa-mem (version 0.7.17)^69^. Downstream analyses for variant calling were performed using SAMtools (version 1.4.1)^70^, Picard Tools MarkDuplicates (version 2.24.0) (http://broadinstitute.github.io/picard/), and GATK HaplotypeCaller (version 4.2.0.0) (Genome Analysis Toolkit) software tools^71,72^. SnpEff (version 5.0e) was used to annotate the variants and their potential mutational effects on associated transcripts^73^; in addition, the loss-of-function mutations, such as splice acceptor/donor site mutations, start codon loss mutations, and stop codon gain/loss mutations, were obtained. Gene annotation model was based on NCBI (release 103) and Ensembl (release 104).

## Supplementary Information

Supplementary Information.

## Data Availability

All data needed to evaluate the conclusions in the paper are present in the paper and the Supplementary Information.
