# Supplementary material for: Cognitive genomics of learning delay and low level of social performance monitoring in macaque
Source: Sci Rep. 2022 Oct 3;12:16539. doi: 10.1038/s41598-022-20948-4 (PMC9529886; doi:10.1038/s41598-022-20948-4)
Supplement: Supplementary file 1 — Supplementary Information. [file 41598_2022_20948_MOESM1_ESM.pdf]

Supplementary information for

Cognitive genomics of learning delay and low level of social performance monitoring in macaque

Taihei Ninomiya, Atsushi Noritake, Shoji Tatsumoto, Yasuhiro Go, Masaki Isoda

	Self	Partner	Mirror	Value	Total reward-related neurons	Total neurons sampled
M1140	138 (12.4)	46 (4.1)	5 (0.5)	6 (0.5)	195 (17.6)	1109
M1969	54 (5.4)	41 (4.1)	2 (0.2)	4 (0.4)	101 (10.1)	1002
M639	12 (1.5)††,**	22 (2.8)	0 (0.0)	0 (0.0)	34 (4.3)††,**	783

Table S1. Number of reward-related neurons in MPFC (early epoch). Comparison between the control (M1140 and M1969) and case (M639) monkeys. Values in parentheses denote the percentage of total neurons sampled. $^{\dagger\dagger}P < 0.01$, M1140 vs. M639; $^{**}P < 0.01$, M1969 vs. M639; chi-square test.

	Self	Partner	Mirror	Value	Total reward-related neurons	Total neurons sampled
M1140	107 (9.6)	167 (15.1)	43 (3.9)	14 (1.3)	331 (29.8)	1109
M1969	88 (8.8)	122 (12.2)	6 (0.6)	10 (1.0)	226 (22.6)	1002
M639	23 (2.9)††,**	61 (7.8)††,**	3 (0.4)††	1 (0.1)†,*	88 (11.2)††,**	783

Table S2. Number of reward-related neurons in MPFC (late epoch). Comparison between the control (M1140 and M1969) and case (M639) monkeys. Values in parentheses denote the percentage of total neurons sampled. $^{†}P < 0.05$, $^{††}P < 0.01$, M1140 vs. M639; $^{*}P < 0.05$, $^{**}P < 0.01$, M1969 vs. M639; chi-square test.

[NCBI]						Case		
**Gene**	**Chr**	**Position**	**LoF type**	**Reference**	**Alt**	**M593**	**M639**	**M344**
COQ2	5	50376380	FRAMESHIFT VARIANT	AAGGAT	A		Hetero	
TCERG1	6	143933953	SPLICE SITE ACCEPTOR	G	A			Hetero
CHRNA5	7	55825070	STOP GAINED	G	T		Hetero	
APTX	15	50884709	STOP GAINED	G	A			Hetero
MVP	20	27521872	STOP GAINED	G	A	Hetero		
UPF3B	X	116143714	SPLICE SITE DONOR	A	T		Hemi	
UPF3B	X	116143715	SPLICE SITE DONOR	C	T		Hemi	

[Ensembl]						Case		
**Gene**	**Chr**	**Position**	**LoF type**	**Reference**	**Alt**	**M593**	**M639**	**M344**
TCERG1 (ENSMMUG00000023288)	6	143933953	SPLICE SITE ACCEPTOR	G	A			Hetero
CHRNA5 (ENSMMUG00000010743)	7	55825070	STOP GAINED	G	T		Hetero	
CLU (ENSMMUG00000021516)	8	27868838	STOP GAINED	G	A		Hetero	
SNCG (ENSMMUG00000011849)	9	51065883	FRAMESHIFT VARIANT	GA	G			Hetero
APTX (ENSMMUG00000007724)	15	50884709	STOP GAINED	G	A			Hetero
DLG4 (ENSMMUG00000010536)	16	6959003	FRAMESHIFT VARIANT	CAG	C		Hetero	
MAPT (ENSMMUG00000004122)	16	57804635	STOP GAINED	G	A	Hetero		
MVP (ENSMMUG00000016422)	20	27521872	STOP GAINED	G	A	Hetero		
UPF3B (ENSMMUG00000020222)	X	116143714	SPLICE SITE DONOR	A	T		Hemi	
UPF3B (ENSMMUG00000020222)	X	116143715	SPLICE SITE DONOR	C	T		Hemi	

Table S3. Loss-of-function mutations found individually in M593, M639, and M344. The coordinates are based on NCBI release 103 (upper) and Ensembl release 104 (lower). Chr, chromosome. Alt, alternate.

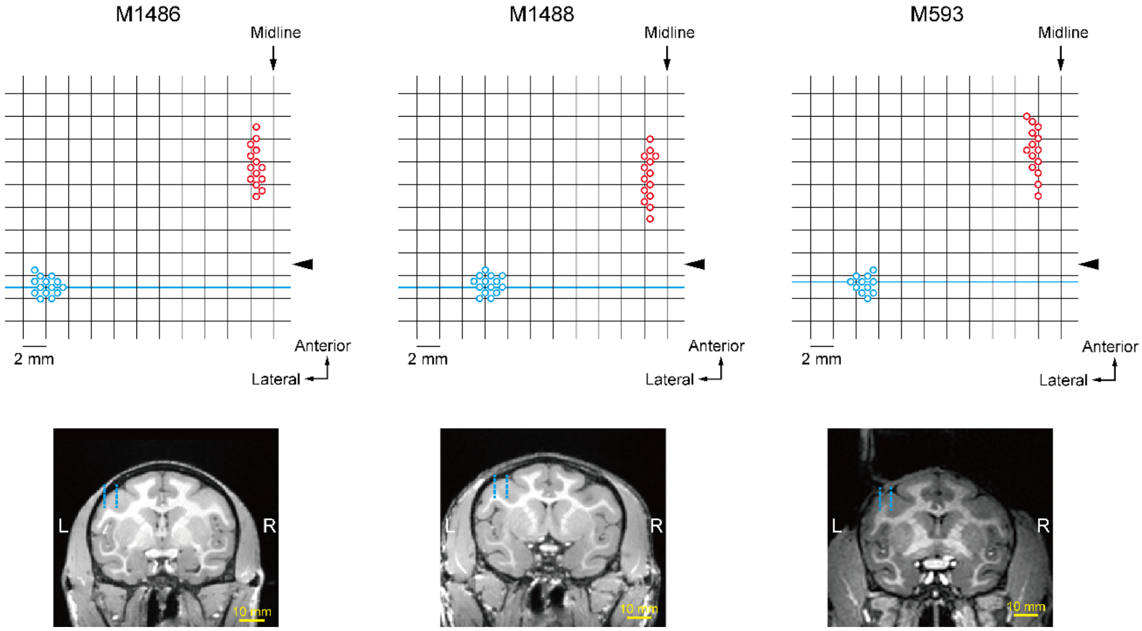

Figure S1. Cortical recording sites for M1486, M1488, and M593. Open circles represent the locations of electrode penetrations (red, MPFC; blue, PMv). Horizontal arrowheads denote the anterior-most locations of the face representation in the supplementary motor area. Horizontal blue lines indicate the approximate anteroposterior level of the MR images on the bottom. The mediolateral extent of recording sites in the PMv is shown by blue vertical dashed lines on the MR images. L, left; R, right.

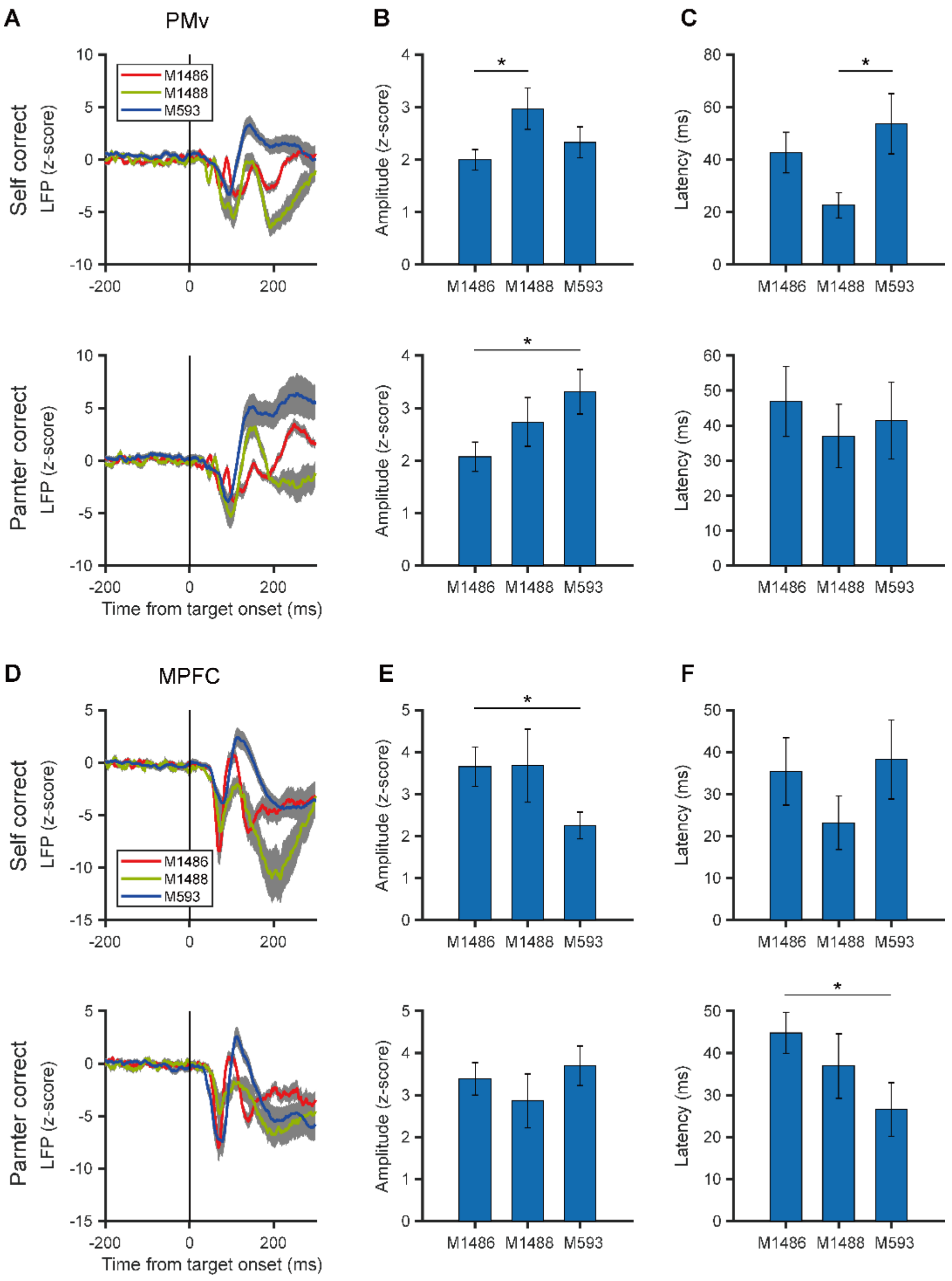

Figure S2. LFP amplitude and latency in M593 and its control. (**A**) Responses of PMv LFPs in correct self-action trials (top) and correct partner-action trials (bottom). (**B**) Rectified amplitude of LFP responses in the PMv. Mean ± SEM. *$P < 0.05$, two-tailed Welch's *t*-test. (**C**) Latency of LFP responses in the PMv. Mean ± SEM. *$P < 0.05$, two-tailed Welch's *t*-test. (**D, E, F**) Responses of MPFC LFPs. Same conventions as in (A), (B), and (C), respectively.

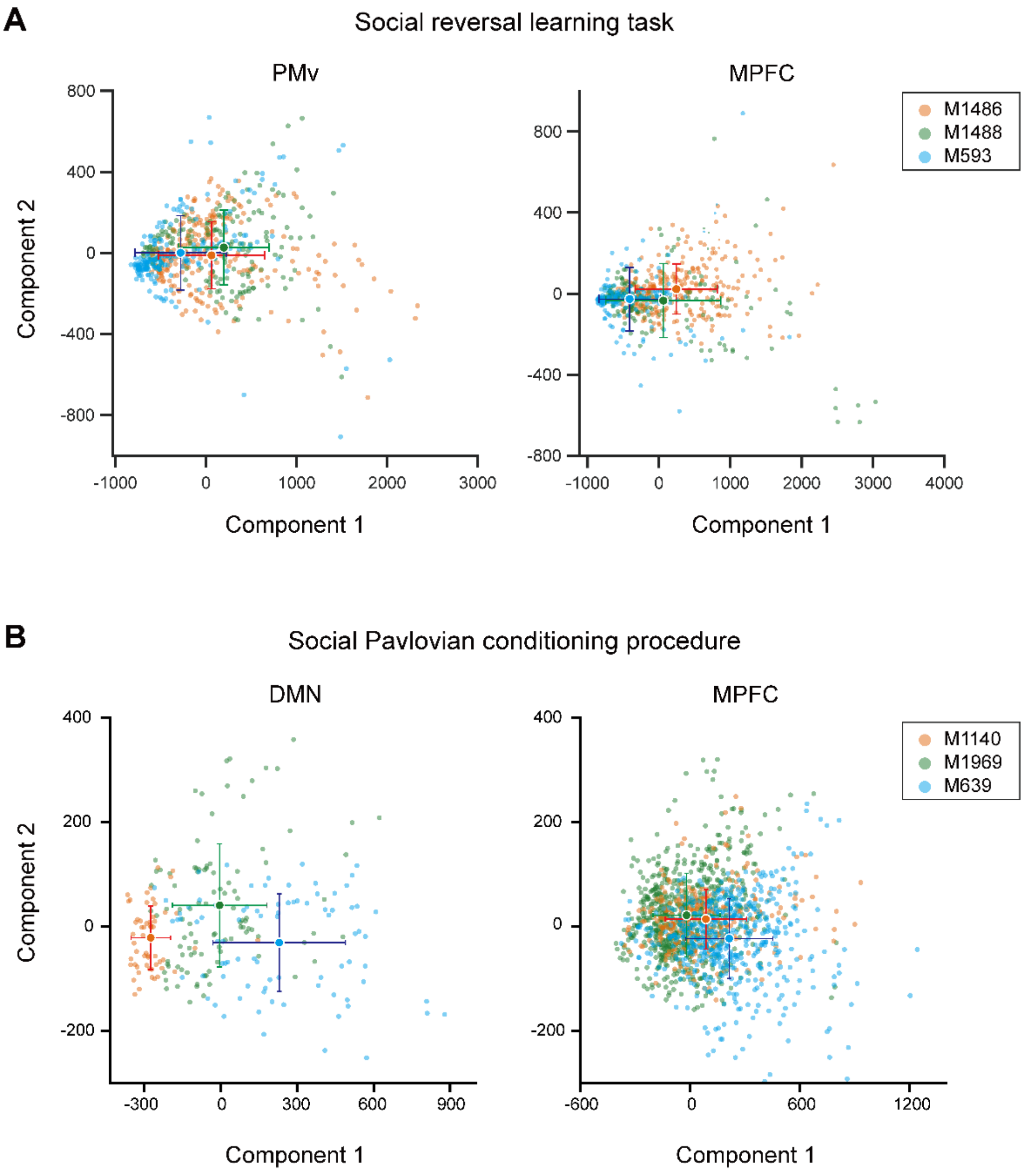

Figure S3. PCA results. (**A**) Data obtained from LFP amplitude and latency during the social reversal learning task. Each small dot represents data derived from one electrode contact during one recording session. Circles indicate the mean values. Error bars indicate standard errors. (**B**) Data obtained from LFP amplitude and latency during the social Pavlovian conditioning procedures. Same conventions as in (A).

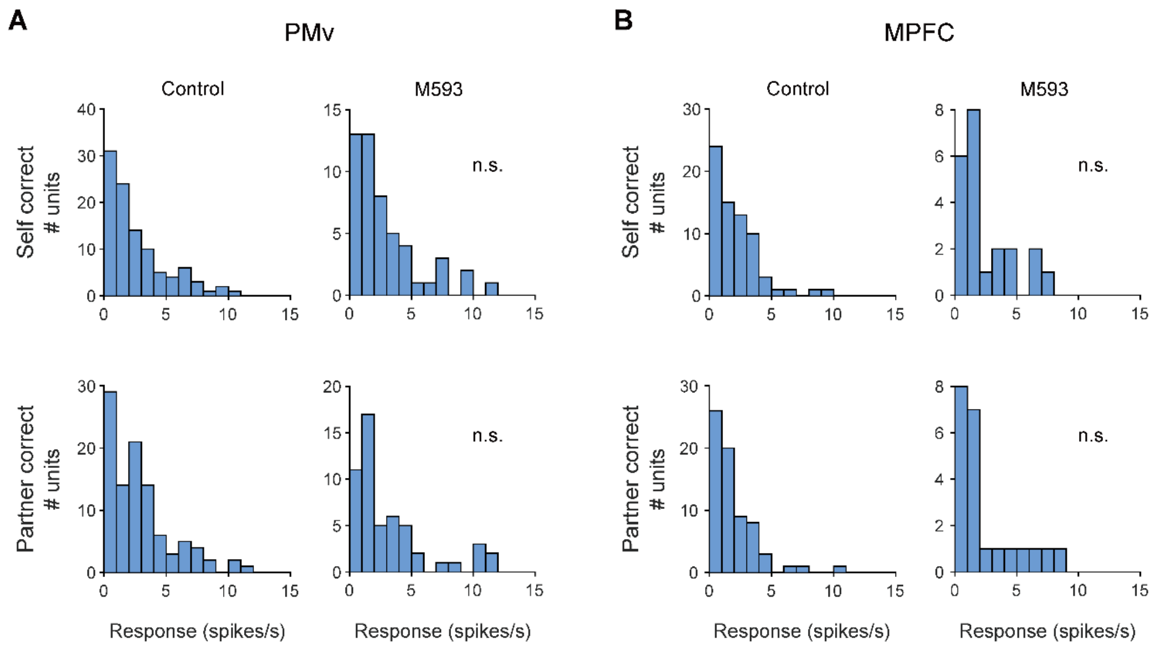

Figure S4. Comparisons of response modulation for mirror-type neurons between M593 and control monkeys (M1486 and M1488). Response in the abscissa indicates the average firing rate during the peri-action period (from 400 ms before to 200 ms after the target button was pressed) *minus* the average firing rate during the control period (0–600 ms before target onset). n.s., not significant between the control monkeys and M593 (two-tailed Welch's *t*-test).

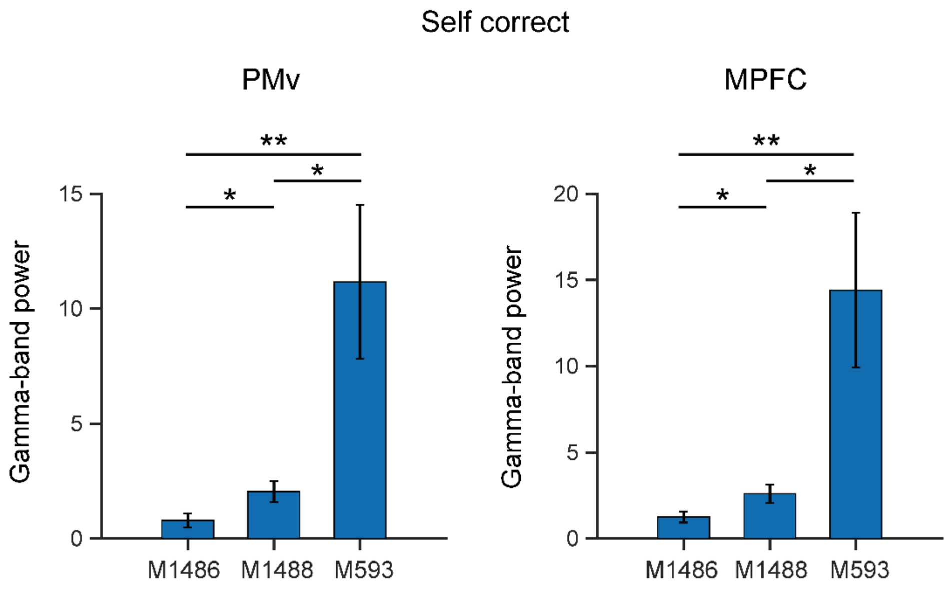

Figure S5. Increased gamma-band (31–55 Hz) power in M593 during self-actions. Mean ± SEM. **P* < 0.05, ***P* < 0.01, two-tailed Welch's *t*-test.

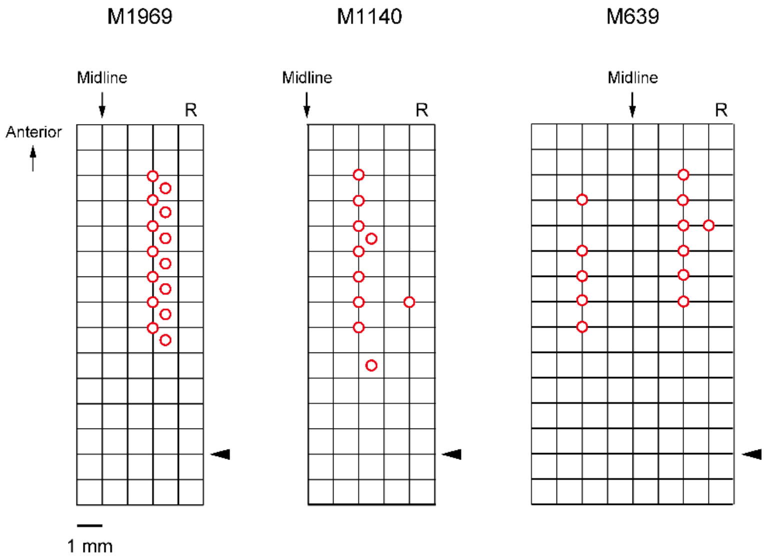

Figure S6. Cortical recording sites for M1969, M1140, and M639. Open red circles represent the locations of electrode penetrations. Horizontal arrowheads denote the anterior-most locations of the face representation in the supplementary motor area. R, right hemisphere.

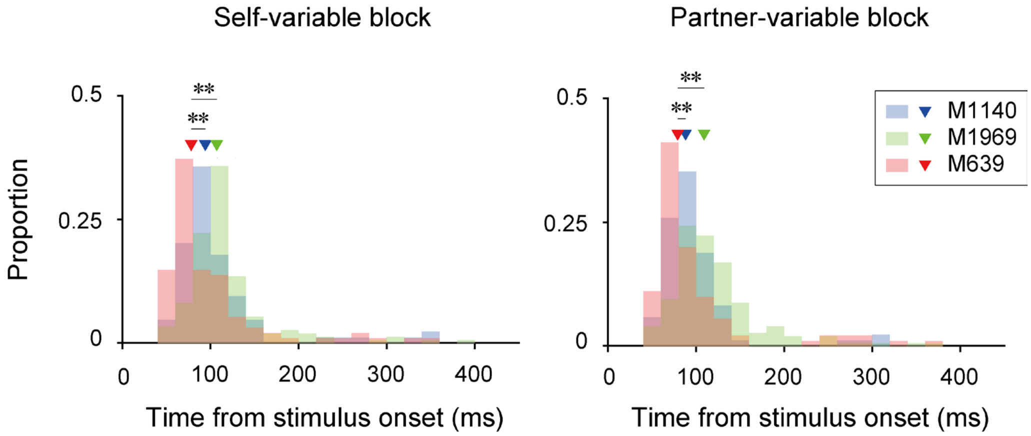

Figure S7. Response latency of dopamine neurons. Latency of peak responses was measured during 51–450 ms after stimulus onset. $^{**}P < 0.01$, Wilcoxon rank sum test. Inverted triangles represent median latencies.

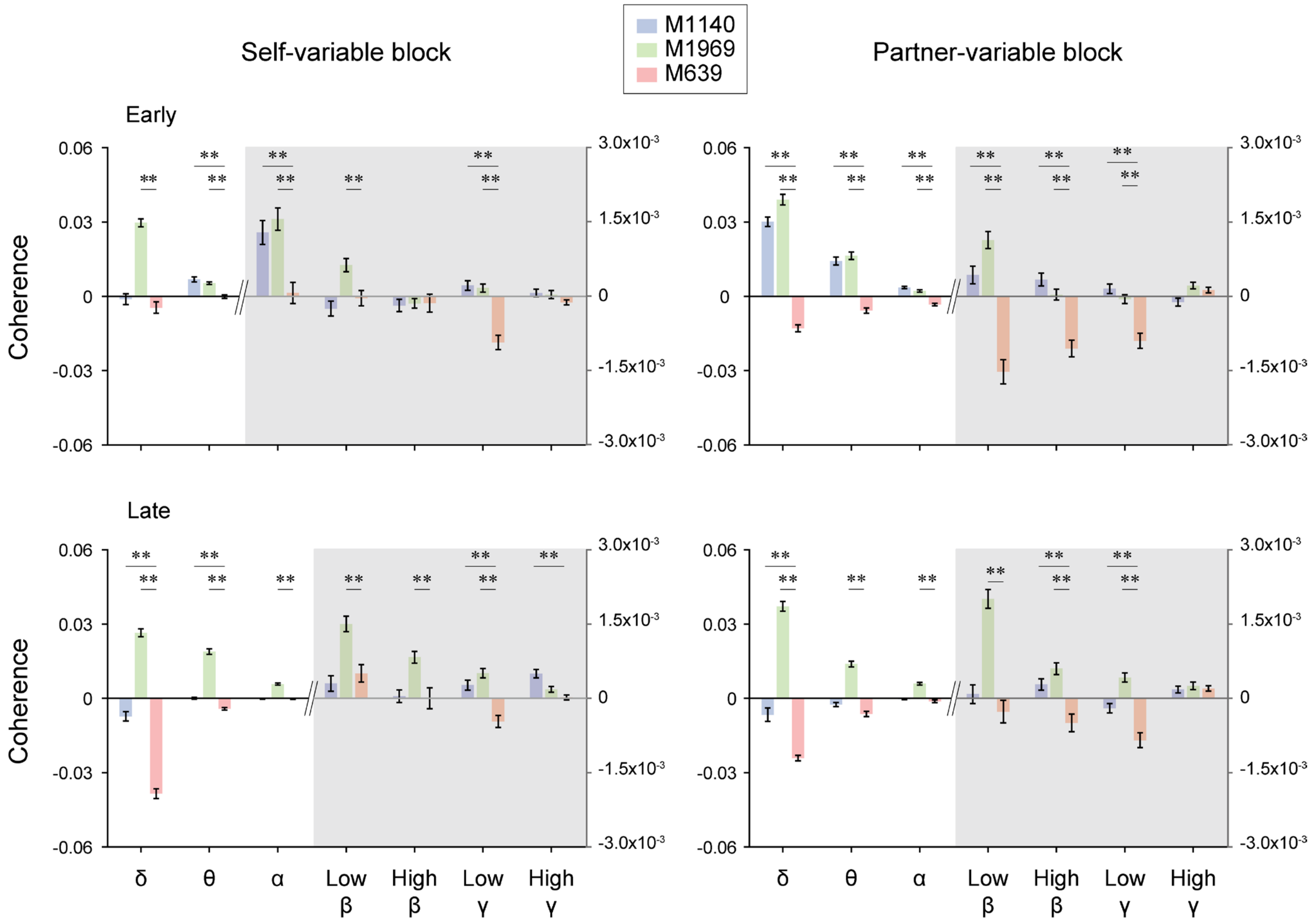

Figure S8. Coherence magnitude at different frequency bands. Mean ± SEM. δ, 1–3 Hz; θ, 4–7 Hz; α, 8–12 Hz; low β, 13–20 Hz; high β, 21–30 Hz; low γ, 31–49 Hz; high γ, 50–128 Hz. $^{**}P < 0.01$, two-tailed Welch's *t*-test with Holm-Bonferroni correction for two task epochs and seven frequency bands. Note that the right ordinate applies to histograms shown in gray shaded areas.
